# Paracrine Orchestration of Tumor Microenvironment Remodeling Induced by GLO1 Potentiates Lymph Node Metastasis in Breast Cancer

**DOI:** 10.1002/advs.202500722

**Published:** 2025-06-10

**Authors:** Jindong Xie, Wenjian Liu, Xinpei Deng, Huan Wang, Xueqi Ou, Xin An, Min‐Yi Situ, Anli Yang, Chuan Peng, Rongfang He, Yi Xie, Hailin Tang, Yuman Chen, Jie‐Ying Liang, Ruonan Shao, Weikai Xiao, Shaoquan Zheng

**Affiliations:** ^1^ State Key Laboratory of Oncology in South China Guangdong Provincial Clinical Research Center for Cancer Sun Yat‐sen University Cancer Center Guangzhou 510060 China; ^2^ The First Affiliated Hospital Hengyang Medical School University of South China Hengyang 421001 China; ^3^ Department of Breast Cancer Guangdong Provincial People's Hospital and Guangdong Academy of Medical Sciences Southern Medical University Guangzhou China; ^4^ Department of Breast Surgery Breast Disease Center The First Affiliated Hospital Sun Yat‐sen University Guangzhou China

**Keywords:** breast cancer, GLO1, lymph node metastases, multi‐omics, tumor microenvironment

## Abstract

Breast cancer is the most prevalent form of malignant tumor that frequently metastasizes to axillary lymph nodes (LNs). Nonetheless, the precise mechanisms underlying alterations in the tumor microenvironment (TME) in LN metastasis in breast cancer remain poorly understood. Single‐cell RNA sequencing of 28 LN samples from 23 patients is performed, and a comprehensive landscape of the entire ecosystem is generated. Ten major cell types are identified, with the subclusters of each major cell type exhibiting diverse characteristics. Furthermore, multiple signatures are collected to evaluate the key components of the subclusters using multi‐omics methodologies. This study finds that myCAFs may hasten LN metastasis, and observed a notable increase in *APOE*+ macrophages and a higher proportion of exhausted *CD8*+ T cells, contributing to the immunosuppressive TME. Moreover, cancer cells in metastatic lesions exhibited diverse expression patterns linked to proliferation, metastasis, oxidative phosphorylation, hypoxia, and interferon responses. Using multi‐omics approaches and experimental validations, it determines that *GLO1* can promote lymphatic angiogenesis, metastasis, and inhibit the proteasomal degradation of *GSS*, thereby maintaining intracellular glutathione (GSH) and reactive oxygen species (ROS) balance. Collectively, the study offers novel perspectives on the microenvironment remodeling of breast cancer LN metastases, suggesting that *GLO1* may be a promising therapeutic target.

## Graphical abstract

Single‐cell RNA sequencing of lymph node (LN) samples is performed, and a comprehensive landscape of the entire ecosystem is generated. Paracrine orchestration of TME remodeling induced by *GLO1* potentiates LN metastasis in breast cancer. This study offers novel perspectives on the microenvironment remodeling of breast cancer LN metastases, suggesting that *GLO1* might be a promising therapeutic target.

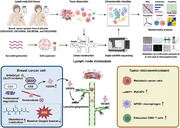

## 1 Introduction

Breast cancer has emerged as a predominant malignancy worldwide, accounting for ≈30% of all cancers in women.^[^ ^1^ ^]^ Owing to rapid advancements in therapeutic strategies, most patients diagnosed with non‐metastatic breast cancer have a high likelihood of being cured. Nevertheless, breast cancer progressing to distant metastasis is regarded as untreatable.^[^ ^2^, ^3^, ^4^ ^]^ The primary modes of metastasis are hematogenous and lymphatic. As the initial site of breast cancer metastasis, the axillary lymph nodes (LNs) are the primary sites of host immune surveillance.^[^ ^5^ ^]^ Clinically, the presence of LN metastasis affects the survival outcomes of patients with breast cancer.^[^ ^6^ ^]^ Biologically, LN metastasis is an ongoing pathological process, and the invasion of tumor cells into LNs is a prerequisite for metastasis. Tumor cells can move toward and infiltrate lymphatic vessels by detecting the chemokine gradients generated by endothelial and myeloid cells.^[^ ^7^, ^8^ ^]^ Additionally, *VEGF* family members (*VEGFA*, *VEGFB*, *VEGFC*, and *VEGFD*) secreted by tumor and stromal cells can promote lymphangiogenesis, thereby increasing pathways to lymphatic vessels.^[^ ^9^, ^10^, ^11^, ^12^ ^]^ Furthermore, the transformation of the LN microenvironment by tumor cells is also a vital aspect. Either breast cancer cells trigger an immune response, leading to cellular rejection for the patient to survive, or the metastatic tumor activates mechanisms to evade immune surveillance.^[^ ^13^, ^14^, ^15^ ^]^ Therefore, an in‐depth investigation on the molecular mechanisms underlying LN metastasis in breast cancer is important to improve patient prognosis.

Genomic and transcriptomic technologies aid in investigating changes in tumor cells during metastasis.^[^ ^16^, ^17^ ^]^ Single‐cell RNA sequencing (scRNA‐seq) provides detailed expression profiles of human cancers at the individual cell level, enabling the identification and description of distinct subclusters with distinctive biological properties.^[^ ^18^, ^19^ ^]^ scRNA‐seq has been extensively utilized in breast cancer studies to investigate the tumor microenvironment (TME) and tumor cell evolution.^[^ ^20^, ^21^, ^22^, ^23^ ^]^ While the majority of studies on LN metastasis in breast cancer have focused on clinical aspects, confirming its risks, few have explored the precise characteristics and mechanisms of different cell types or the remodeling of the LN microenvironment by cancer cells.^[^ ^24^, ^25^, ^26^, ^27^ ^]^ Besides, although LNs are rich in immune cells, they can still harbor cancer cells that can survive and proliferate within the TME, prompting further investigation into TME components and their potential role in either creating a favorable environment for metastatic cancer cells or transitioning into an immunosuppressive state.

**Figure 1:**
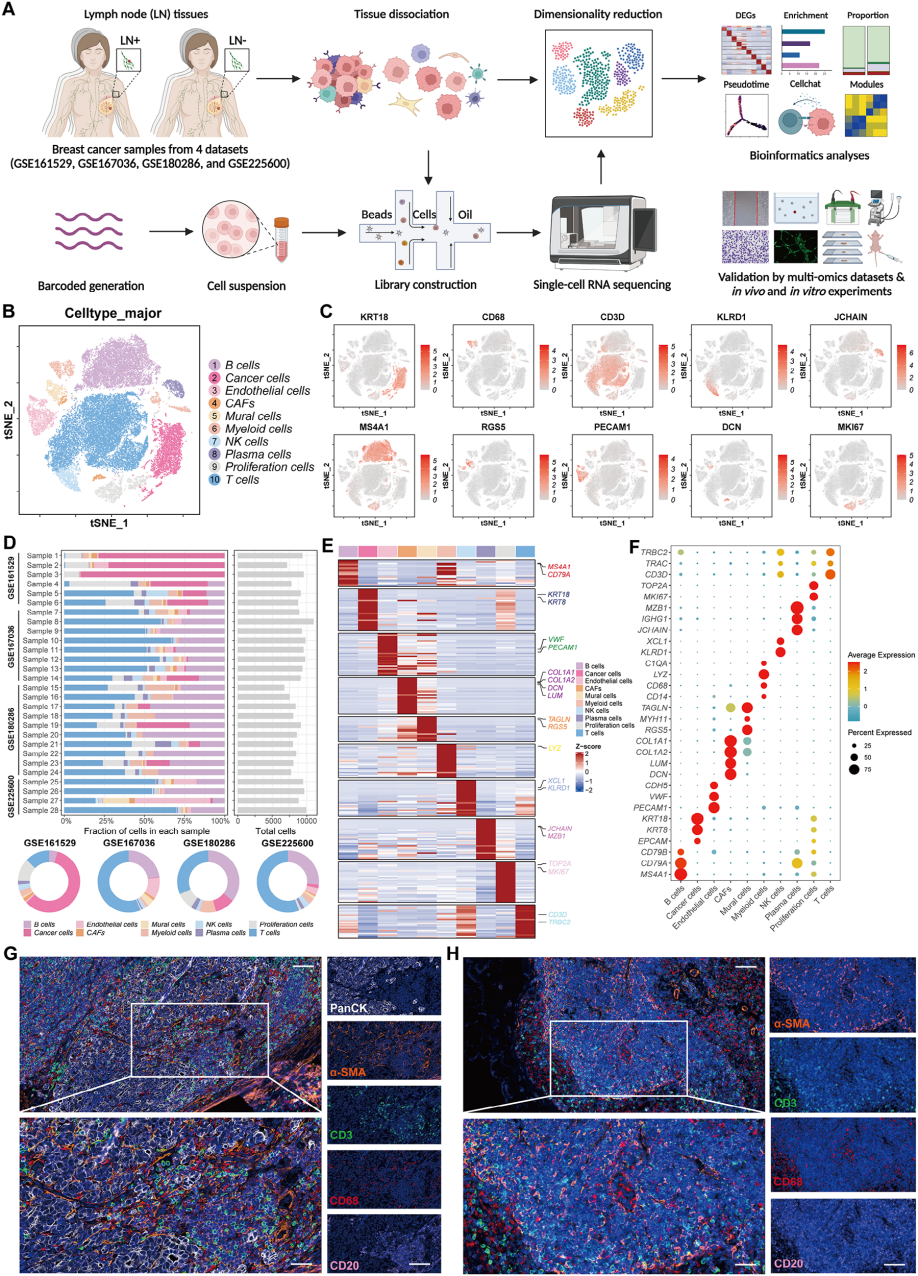
Single‑cell atlas of LN metastasis in breast cancer. A) Overview of the study design and workflow. 28 LN samples (23 with metastases and 5 with non‐metastases) from 23 patients were collected from four datasets (created with BioRender.com). B) t‐SNE plot of single cells profiled in the present study colored by major cell type. C) Feature plots for the canonical marker genes of cancer cells (*KRT18*), B cells (*MS4A1*), myeloid cells (*CD68*), mural cells (*RGS5*), T cells (*CD3D*), endothelial cells (*PECAM1*), NK cells (*KLRD1*), CAFs (*DCN*), plasma cells (*JCHAIN*), and proliferation cells (*MKI67*). D) Bar plots showing the relative proportions of each major cell subtype in each sample and dataset. E) Heatmap showing the expression levels of classic marker genes across all major cell types. F) Dot plot showing the expression levels of classic marker genes across all major cell types. G,H) Representative images of multiplex immunofluorescent staining of breast cancer LN tissues with (G) and without (H) metastasis.

The present study aimed to explore the regulatory mechanisms of the TME within LNs that could potentially influence cancer cell metastasis and colonization and to distinguish LN tissues with metastases from those without through scRNA‐seq. Ten major cell types were identified in 28 LN samples, with the subclusters of each major cell type exhibiting diverse characteristics. Furthermore, by integrating multi‐omics bioinformatics analyses with in vitro and in vivo experiments, we determined that *GLO1* might facilitate LN metastasis. This study offers new profound insights into the LN metastasis of breast cancer cells, investigate the remodeling process of the LN microenvironment, and elucidate the role of *GLO1* as a promising biomarker and therapeutic target for improving the treatment outcomes of patients with breast cancer. **Figure** **1**A shows a schematic of the study design and workflow.

## 2 Results

### 2.1 Single‑Cell Atlas of LN Metastasis in Breast Cancer

The composition of LN metastases lesions in breast cancer was analyzed using scRNA‐seq. Following the quality control measures, doublet elimination, unsupervised clustering, and copy number variation (CNV) analysis, several clusters of cells displaying comparable expression patterns were identified (Figure 1B; Figure S1A–D, Supporting Information). By using classic markers, each cluster was verified to be a distinct cell population: B cells (expressing MS4A1 and CD79B), cancer cells (expressing *KRT18* and *KRT8*), endothelial cells (ECs; expressing *PECAM1* and *VWF*), cancer‐associated fibroblasts (CAFs, expressing *DCN* and *LUM*), mural cells (expressing *RGS5* and *TAGLN*), myeloid cells (expressing *LYZ* and *CD68*), natural killer cells (NKs; expressing *KLRD1* and *XCL1*), plasma cells (expressing *JCHAIN* and *MZB1*), proliferation cells (expressing *MKI67* and *TOP2A*), and T cells (expressing *CD3D* and *TRBC2*) (Figure 1C; Figure S1E, Supporting Information). Besides, the distribution of the major cell subtypes among the samples and datasets is shown in Figure 1D. Heatmap and dot plots showing cluster‐specific markers according to their expression levels and proportions are presented in Figure 1E,F. We also conducted multiplex immunofluorescent (mIF) staining to verify cell subpopulations in metastatic LN (LN+) and non‐metastatic LN (LN‐) tissues in breast cancer (Figure 1G,H).

### 2.2 Cell Clustering and Categorization of Stromal Cells in Breast Cancer LN Tissues

**Figure 2:**
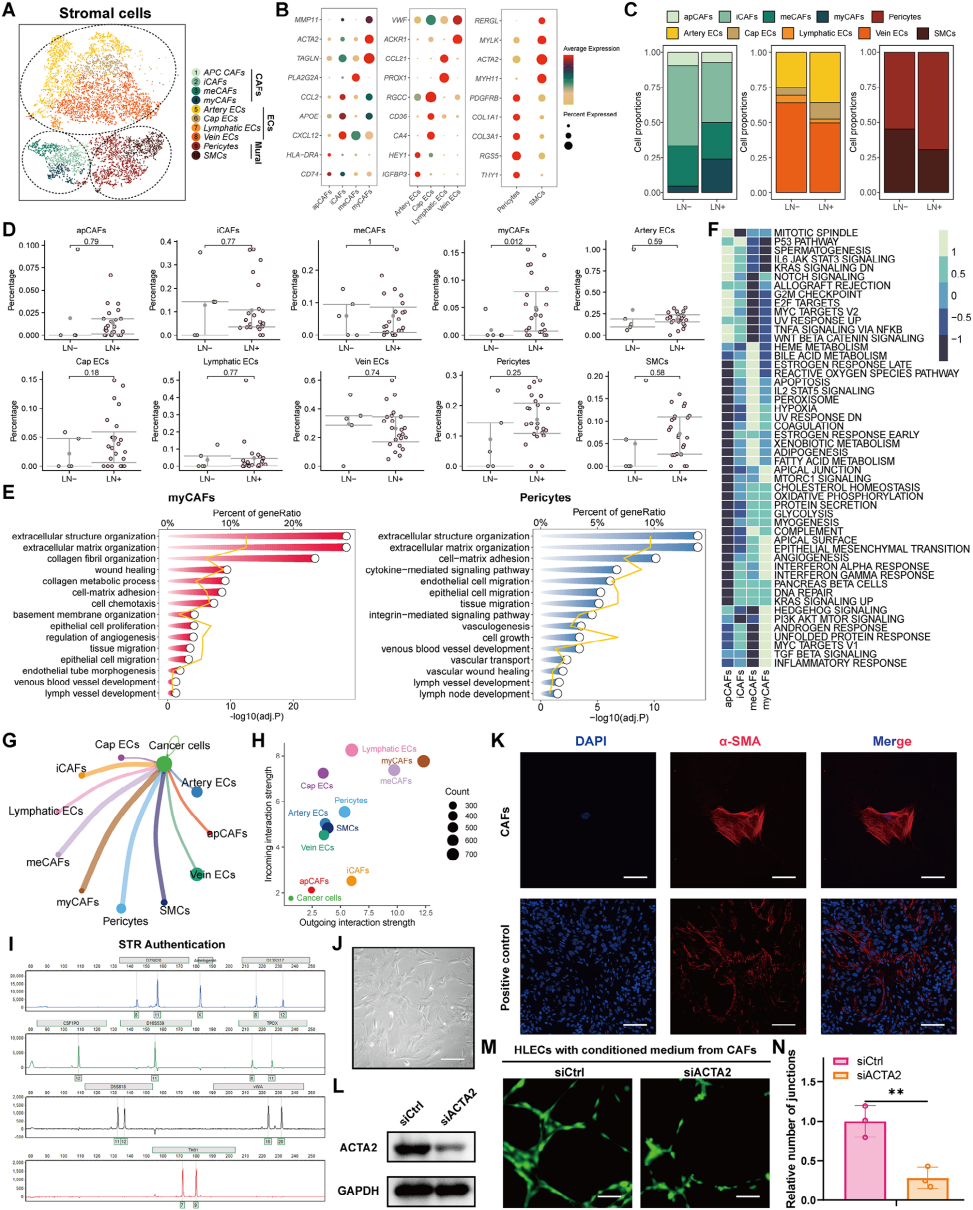
Cell clustering and categorization of stromal cells in breast cancer LN tissues. A) t‐SNE plot of the stromal cells (CAFs, ECs, and mural cells) landscape. B) Dot plots showing the expression levels of classic markers in each stromal cell subcluster. C) Bar plot for cell proportion of stromal cell clusters between LN‐ and LN+ tissues. D) Scatter plots for the different percentage of each stromal cell subcluster between LN‐ and LN+ tissues. E) Pathway enrichment analyses of myCAFs and pericytes using Gene Ontology (GO) database. F) Heatmap showing the activated pathways and biological processes among CAFs subclusters using hallmark gene sets. G) Cellchat analyses showing number of interactions among different cell types. H) Outgoing and incoming interaction strength between cancer cells and stromal cell subclusters. I) STR authentication of patient‐derived CAFs. J) Representative bright filed image of patient‐derived CAFs. K) Immunofluorescent staining verifies the reliability of patient‐derived CAFs using classic myCAFs biomarker *α‐SMA* (encoded by *ACTA2*). L) Western blotting confirming the knockdown efficacy of *ACTA2* in CAFs. M) Tube formation assays using HLECs treated with conditioned medium from CAFs treated with siCtrl and siACTA2. N) Histogram showing relative number of junctions between CAFs treated with siCtrl and siACTA2. (*** means p < 0.001).

The stromal compartment primarily comprised CAFs, ECs, and mural cells. CAFs were regrouped into four subclusters based on the most variable genes and classic markers: antigen‐presenting CAFs (apCAFs, expressing *CD74* and *HLA‐DRA*); inflammatory CAFs (iCAFs, expressing *CXCL12*, *APOE*, and *CCL2*); metabolic CAFs (meCAFs, expressing *PLA2G2A*); myofibroblastic CAFs (myCAFs, expressing *TAGLN*, *ACTA2*, and *MMP11*). ECs were re‐clustered into four subclusters: artery ECs (expressing *IGFBP3* and *HEY1*); capillary ECs (Cap ECs, expressing *CA4*, *CD36*, and *RGCC*); lymphatic ECs (expressing *PROX1* and *CCL21*); vein ECs (expressing *ACKR1* and *VWF*). Mural cells were re‐clustered into two subclusters: Pericytes (expressing *THY11*, *RGS5*, *COL3A1*, *COL1A1*, and *PDGFRB*); smooth muscle cells (SMCs, expressing *MYH11*, *ACTA2*, *MYLK*, and *RERGL*) (**Figure** **2**A,B; Figure S2A–C, Supporting Information).

Subsequently, the cell proportions of each subtype were compared between the LN‐ and LN+ groups. The results indicated that that the LN+ group had a notably higher proportion of the myCAFs subcluster than the LN‐ group (Figure 2C,D). Additionally, enrichment analyses revealed that myCAFs and pericytes participated in collagen fibril organization, lymph vessel development, extracellular structure organization, and wound healing; apCAFs were associated with antigen‐presenting processes and immune cells homeostasis and differentiation; iCAFs were related to cell chemotaxis and factor secretion; meCAFs were responsible for metabolic processes and ATP generation; SMCs contributed to muscle cell proliferation, development, and contraction (Figure 2E; Figure S2D, Supporting Information). Moreover, we calculated the activity scores using the hallmark gene set and found that myCAFs exhibited high activity in EMT, angiogenesis, and interferon responses (Figure 2F). Furthermore, we performed cell‐cell communication analyses, and the results showed that myCAFs had the most interactions with cancer cells among the stromal cell subclusters (Figure 2G). Additionally, myCAFs exhibited the strongest interaction strength, whereas lymphatic ECs had the highest incoming strength (Figure 2H). These findings indicated that myCAFs might play a vital role in LN metastasis in breast cancer.

To verify our hypothesis, we collected and extracted breast cancer patient‐derived CAFs and subsequently confirmed their reliability via short tandem repeat (STR) authentication and IF staining (Figure 2I–K). As *ACTA2* is a classic marker for myCAFs, we designed an siRNA targeting *ACTA2* and validated its efficacy by Western blotting analysis (Figure 2L). Human lymphatic endothelial cells (HLECs) tube formation assays showed that *ACTA2* knockdown in CAFs significantly inhibited tube formation (Figure 2M,N). Furthermore, ligand‐receptor analysis showed that *COL1A1/COL1A2*‐*SDC4/CD44* and *MDK/NCL* presented the highest communication probability, suggesting that these ligand‐receptor pairs are necessary for LN metastasis in breast cancer (Figure S2E,F, Supporting Information).

### 2.3 Characterization of Immunosuppressive Myeloid Cells in Breast Cancer LN Tissues

**Figure 3:**
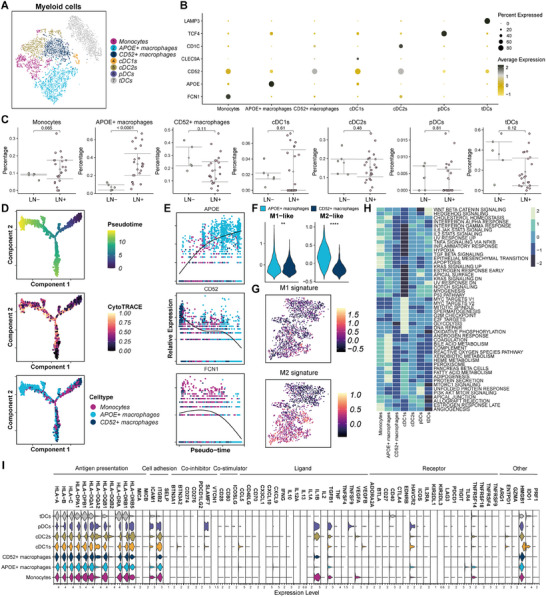
Characterization of immunosuppressive myeloid cells in breast cancer LN tissues. A) t‐SNE plot of the myeloid cells (monocytes, macrophages, and DC cells) landscape. B) Dot plots showing the expression levels of classic markers in each myeloid cell subcluster. C) Scatter plots for the different percentage of each myeloid cell subcluster between LN‐ and LN+ tissues. D) Pseudo‐time trajectory of monocytes and macrophages subclusters by Monocle2. E) The expression of *APOE*, *CD52*, and *FCN1* involved in the cell state transition. F) Violin plots showing the different levels of M1 and M2 signature score between *APOE*+ macrophage and *CD52*+ macrophage cell subclusters. G) Feature plots showing the distribution of M1 and M2 signature scores. H) Heatmap showing the activated pathways and biological processes among myeloid cell subclusters using hallmark gene sets. I) Violin plots showing the scaled immune‐related genes expression among myeloid cell subtypes subclusters.

We examined the behavior of myeloid cells in LN tissues from patients with breast cancer. Monocytes, macrophages, and dendritic cells (DCs) constituted the myeloid cell population, which was further classified into seven distinct clusters (**Figure** **3**A). Macrophages were divided into two subclusters—namely, *APOE*+ macrophages and *CD52*+ macrophages; DCs were divided into four subclusters: conventional DC1s (cDC1s), conventional DC2s (cDC2s), plasmacytoid DCs (pDCs), and tolerogenic DCs (tDCs). Dot and feature plots that graphically present the markers and proportions associated with each subcluster are shown in Figure 3B and Figure S3A (Supporting Information).

We then computed the proportion of each subcluster and observed a significantly elevated proportion of *APOE*+ macrophages in the LN+ group compared with the LN‐ group (Figure 3C; Figure S3B, Supporting Information). Additionally, pseudo‐time trajectory analysis results indicated that *APOE*+ macrophages and *CD52*+ macrophages differentiated in different directions (Figure 3D,E). Besides, collected and calculated the scores for the signatures specific to macrophages and found that the *APOE*+ macrophage subcluster was correlated with M2‐like macrophages, whereas the *CD52*+ macrophage subcluster was similar to M1‐like macrophages (Figure 3F,G). By applying the “scMetabolism” algorithm,^[^ ^28^ ^]^ we evaluated the disparities in metabolic pathways across various subclusters and found that the *APOE*+ macrophage subcluster exhibited an augmentation in metabolic pathway activity. Conversely, the *CD52*+ macrophage subcluster showed a reduction in metabolic pathway activity (Figure S3C, Supporting Information). Moreover, we explored the potential relevance of the signaling pathways and biological functions for each subcluster using hallmark pathway gene sets. The results indicated that the monocyte, *APOE*+ macrophage, and cDC1 subclusters displayed the most pronounced activity across most signaling pathways and biological functions (Figure 3H). Subsequently, we analyzed cell‐cell communication and found that the *APOE*+ macrophage subcluster exhibited extensive communication networks with cancer cells (Figure S3D,E, Supporting Information). Ligand receptor analysis revealed that *APOE*+ macrophages may interact with cancer cells via the *SPP1*‐*CD44* and *FN1*‐*SDC4*/*CD44* signaling pathways, whereas monocytes and DCs may interact with cancer cells via the *LGALS9*‐*CD44* signaling pathway (Figure S3F, Supporting Information). In addition, Figure S3G (Supporting Information) shows the expression levels of the key genes. As myeloid cells are key sources of immune checkpoints within the TME,^[^ ^29^, ^30^ ^]^ classic immune‐related genes were collected, and their expression levels were assessed. The results indicated that the majority of these immune‐related genes exhibited low expression levels, except for *ICAM1*, *ITGB2*, *IL1B*, *TGFB1*, *HMGB1*, and genes associated with antigen presentation (Figure 3I).

### 2.4 T/NK Cells were Distinguished in Breast Cancer LN Tissues

**Figure 4:**
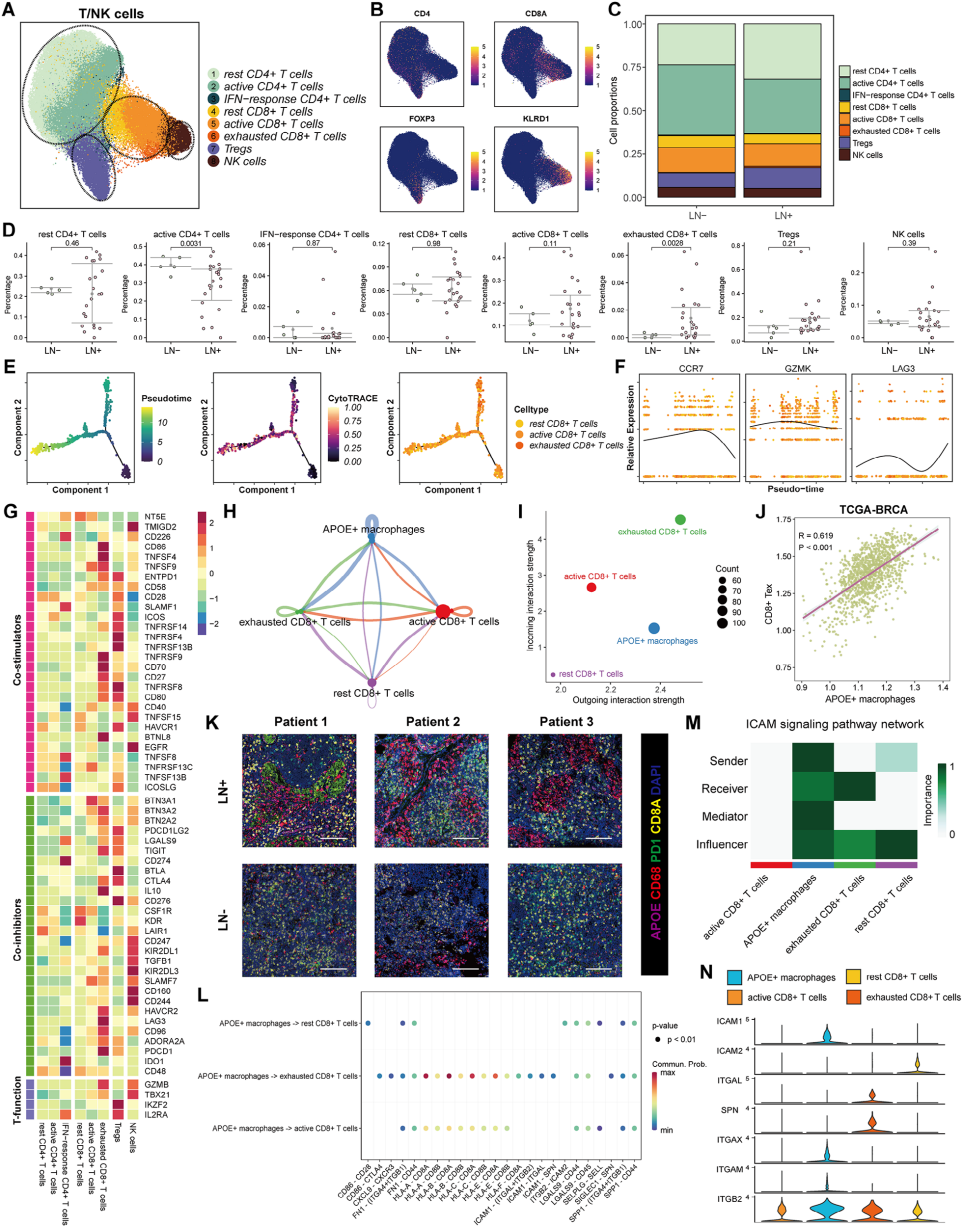
T/NK cells were distinguished in breast cancer LN tissues. A) t‐SNE plot of the T/NK cells landscape. B) Feature plots showing the normalized expression of highly expressed genes in each T/NK cell subcluster. C) Bar plot for cell proportion of T/NK cell clusters between LN‐ and LN+ tissues. D) Scatter plots for the different percentage of each T/NK cell subcluster between LN‐ and LN+ tissues. E) Pseudo‐time trajectory of *CD8*+ T cell subcluster by Monocle2. F) The expression of *CCR7*, *GZMK*, and *LAG3* involved in the cell state transition. G) Heatmap showing the scaled expression levels of co‐stimulators, co‐inhibitors, and T‐function markers among T/NK cells subclusters. H) Cellchat analyses showing number of interactions between *APOE*+ macrophages and *CD8*+ T cell subclusters. I) Outgoing and incoming interaction strength between *APOE*+ macrophages and *CD8*+ T cell subclusters. J) Scatter plots showing the correlation between *APOE*+ macrophages score and *CD8*+Tex score. K) Representative mIF staining images showing the spatial distribution of *CD68*, *APOE*, *CD8A*, and *PD1* in breast cancer LN tissues (n = 3). L). Ligands and receptors for signal communication between APOE+ macrophages and *CD8*+ T cell subclusters. M) Heatmap showing the importance of *APOE*+ Macrophages and *CD8*+ T cell subclusters during *ICAM* signaling pathway network. N) Violin plots showing the expression levels of certain genes in *ICAM* signaling pathway among *APOE*+ macrophages and *CD8*+ T cell subclusters.

A secondary clustering analysis of T/NK cells was conducted to delineate high‐resolution subclusters within breast cancer LN tissues. Eight distinct subclusters of T/NK cells were identified in the entire sample population (**Figure** **4**A,B). Dot plots presenting a comprehensive summary of marker expression across individual subclusters is shown in Figure S4A (Supporting Information). The *CD4*+ T cell cluster comprised resting *CD4*+ T cells, active *CD4*+ T cells, IFN‐response *CD4*+ T cells, and regulatory T cells (Tregs), whereas the *CD8*+ T cell cluster included resting *CD8*+ T cells, active *CD8*+ T cells, and exhausted *CD8*+ T cells.^[^ ^31^ ^]^ Tregs were also clustered into two subclusters: resting Tregs and terminal Tregs (Figure S4B,C, Supporting Information).^[^ ^32^ ^]^ Compared with the LN‐ group, LN+ tissues exhibited a significantly higher proportion of exhausted *CD8*+ T cells but had a lower proportion of active *CD4*+ T cells. These findings suggested that LN+ tissues may be located in an immunosuppressive environment (Figure 4C,D; Figure S4D,E, Supporting Information). Additionally, pseudo‐time trajectory analysis confirmed that active *CD8*+ T cells and exhausted *CD8*+ T cells had divergent differentiation pathways (Figure 4E,F). To evaluate the comprehensive impact of each T/NK cell subcluster, we quantified the expression levels of immune‐related genes involved in co‐stimulation, co‐inhibition, and specific T‐cell functions and found that exhausted *CD8*+ T cells and Treg subclusters exhibited both co‐stimulator and co‐inhibitor signatures (Figure 4G). Given that immune features may substantially vary by subtype, we explored the differences among subtypes and found that the TNBC subtype exhibited the most immunosuppressive features (Figure S4F, Supporting Information). Enrichment analyses were also performed using hallmark gene sets to assess the activity of each CD4+ and CD8+ T‐cell subcluster (Figure S4G, Supporting Information). Given the vital role of the interplay between exhausted *CD8*+ T cells and *APOE*+ macrophages in cancer immunotherapy,^[^ ^33^ ^]^ we conducted a cell‐to‐cell communication analysis to explore whether they interact with each other. The results indicated that exhausted *CD8*+ T cells had the most interactions with *APOE*+ macrophages among the CD8+ T cell subclusters (Figure 4H–J).

Additionally, we established the “*APOE*+ macrophages” and “exhausted *CD8*+ T cells” signatures and then computed their activity scores for each sample across several large‐cohort datasets. In line with our expectations, we found strongly positive correlations between the “*APOE*+ macrophages” and “exhausted *CD8*+ T cells” signatures among these datasets (Figure 4J; Figure S4H, Supporting Information). Furthermore, we collected breast cancer LN tissues and conducted mIF staining, which showed that in LN tissues with metastasis, *APOE*+ macrophages and *CD8*+ exhausted T cells were in close proximity, whereas in those without LN metastasis, these two subpopulations were sparsely distributed (Figure 4K; Figure S4J, Supporting Information). These findings indicate that the TME may be related to the interplay between *APOE*+ macrophages and exhausted *CD8*+ T cells in LN metastasis in breast cancer. Moreover, *APOE*+ macrophages might interact with exhausted *CD8*+ T cells via the *ICAM*, *HLA‐I*, and *CD86* signaling pathways (Figure 4L–N; Figure S4J,K, Supporting Information).

### 2.5 Transcriptomic Diversity of B and Plasma Cells in Breast Cancer LN Tissues

B and plasma cells are integral to adaptive immunity and play crucial roles in both humoral and cellular immunity.^[^ ^34^ ^]^ To analyze the transcriptomic diversity of B/Plasma cells in breast cancer LN tissues, we isolated the cells by re‐clustering distinct B/Plasma‐cell subpopulations initially extracted from the original data. After extracting all B/Plasma cells based on the marker genes *CD79B*, *MS4A1*, *JCHAIN*, and *MZB1*, three distinct B cell subtypes and two distinct plasma cell subtypes were identified. In B cell subpopulations, naïve B cells expressed higher levels of *IL7R*, germinal center (GC) B cells showed *LRMP* enrichment, and memory B cells highly expressed *TNFRSF13B*. As for plasma cells, *IgA*+ plasma cells and *IgG*+ plasma cells were differentiated by their immunoglobulin transcript expression levels (Figure S5A,B, Supporting Information). Although all five B/plasma‐cell subtypes were observed in both LN‐ and LN+ tissues, the degree of infiltration varied among these subtypes, indicating distinct B/plasma cell infiltration patterns at different stages of breast cancer advancement. Comparison between the LN+ and LN‐ groups revealed a significant increase in plasma cells with *IgA*+/*IgG*+ subtypes and a significant decrease in memory B cells (Figure S5C,D, Supporting Information). Pathway enrichment analyses showed that memory B cells were related to chaperone‐mediated processes, cytoplasmic translation, and protein folding, whereas plasma cells exhibited a strong correlation with glycosylation and endoplasmic reticulum stress (ERS) (Figure S5E, Supporting Information). Additionally, hallmark gene set enrichment analysis indicated that B cells were activated by the interferon response and that plasma cells were enriched in the unfolded protein response (a type of adaptation response to ERS), epithelial‐mesenchymal transition, angiogenesis, and glycolysis (Figure S5F, Supporting Information). Moreover, most biological processes mentioned above possessed higher activity in the LN+ group compared with the LN‐ group (Figure S5G, Supporting Information). Furthermore, we utilized the “CellChat” R package to analyze cell‐cell communication and confirmed that the LN+ group had more communication weights with other cell types (Figure S5H,I, Supporting Information). Collectively, our analyses revealed the transcriptomic diversity of B/Plasma subclusters in breast cancer LN tissues.

### 2.6 Discernment of Common Expression Programs of Cancer Cells in Breast Cancer LN Metastatic Lesions

**Figure 5:**
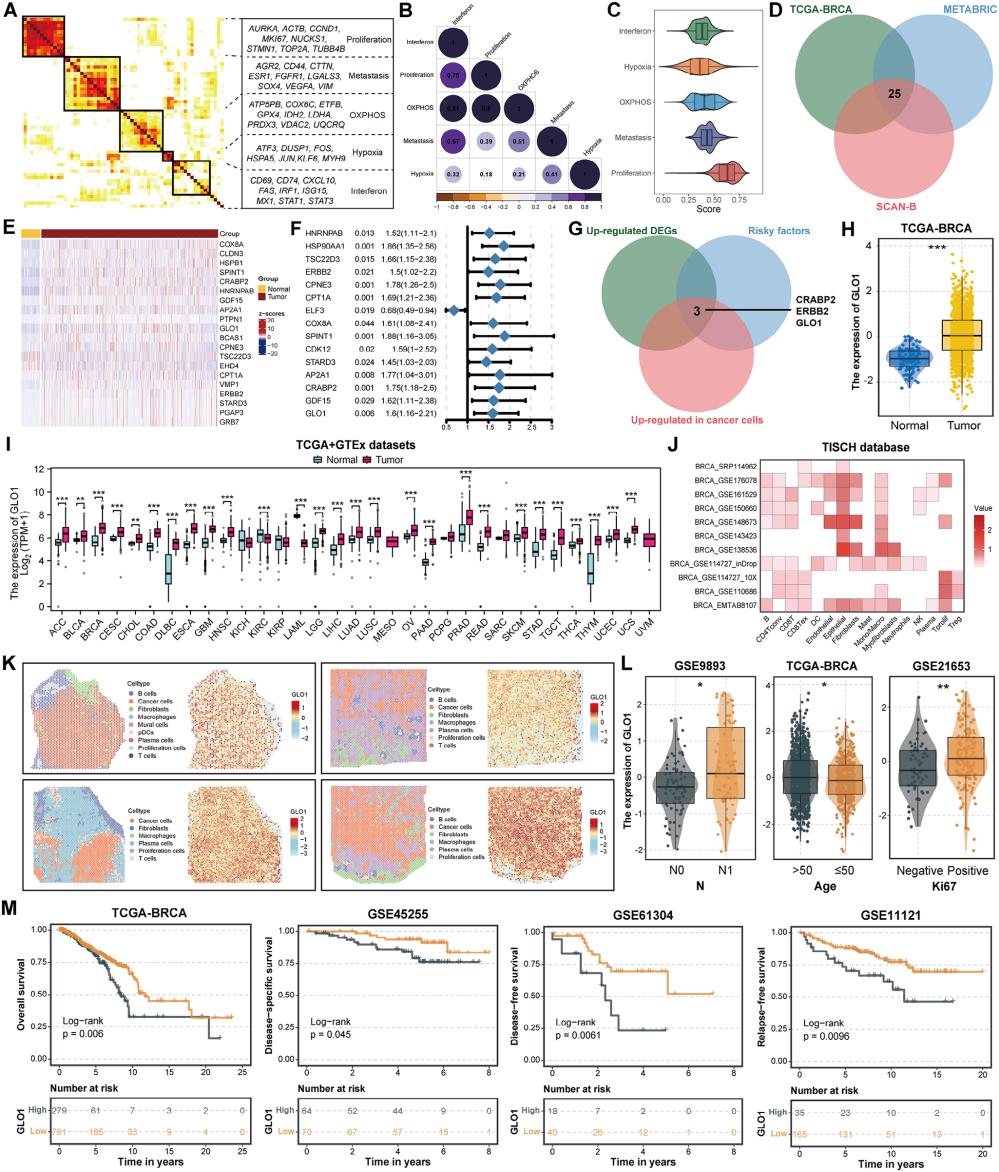
Discernment of common expression programs of cancer cells in breast cancer LN metastatic lesions and identified *GLO1* was a biomarker of LN metastasis in breast cancer. A) Heatmap showing five common programs of cancer cells in breast cancer LN metastatic lesion. B) Heatmap revealing the pairwise interactions among the expression programs. C) Violin plot of the proportions among five expression programs. D) Venn plot showing the intersection differential expression genes (DEGs) of samples with and without LN metastasis between three breast cancer cohorts and the “metastasis” program. E) Heatmap showing DEGs between tumor and normal samples in the TCGA‐BRCA cohort. F) Forest plot showing significant DEGs relevant to overall survival in the TCGA‐BRCA cohort using K‐M survival analysis. G) Venn plot showing the intersection genes following filter criteria: upregulated in tumor samples; risky factor for survival; upregulated in cancer cells. H) Box plot showing the expression of *GLO1* between normal and tumor tissues in TCGA‐BRCA dataset. I) Box plots showing the expression of *GLO1* between tumor and normal tissues in TCGA and GTEx pan‐cancer datasets. J) Heatmap showing the expression and distribution of *GLO1* among breast cancer scRNA‐seq datasets using TISCH database. K) The spatial transcriptomic (ST) clusters are overlaid upon the breast cancer LN metastasis biopsies, and the *GLO1* expression levels are also shown. L) Box plots showing the expression of *GLO1* between different groups in GSE9893, TCGA‐BRCA, and GSE21653 datasets. M) K‐M survival analysis showing the specific role of *GLO1* in overall survival, disease‐specific survival, disease‐free survival, and relapse‐free survival outcomes in different cohorts. (* means p < 0.05, ** means p < 0.01, and *** means p < 0.001).

Subsequently, transcriptome pattern and subpopulation clustering analyses were conducted to elucidate the diversity of cancer cells. A meta‐cluster algorithm was devised to identify common programs among the LN metastatic breast cancer tissues. We conducted a comparative analysis of the most active cancer hallmarks in programmed and non‐programmed cells for each specific program. Our investigation identified five distinct expression programs, each characterized by unique biological functions and cellular states. These programs include proliferation, metastasis, oxidative phosphorylation (OXPHOS), hypoxia, and interferon (**Figure** **5**A; Figure S6A, Supporting Information). The proliferation program demonstrated increased expression levels of cell cycle‐associated genes, including *AURKA*, *TOP2A*, *STMN1*, and *MKI67*; The metastasis program encompassed genes like *AGR2*, *SOX4*, *VIM*, etc. The OXPHOS program consisted of genes such as *COX6C*, *GPX4*, *VDAC2*, and *UQCRQ*; The hypoxia program was characterized by genes like *FOS*, *HSPA5*, *JUN*, and *KLF6*; The interferon program consisted of a series of interferon‐related genes (*ISG15*, *MX1*, *STAT1*, and *STAT3*). Furthermore, pairwise interactions between programs were validated using correlation analysis, and program scores were determined using quantitative analysis of the proportion of program cells corresponding to each program's activity level (Figure 5B). The findings indicated that the “Hypoxia” module generally exhibited low activity (Figure 5C).

### 2.7 *GLO1* is a Biomarker of LN Metastasis in Breast Cancer

We subsequently performed bioinformatic analyses to investigate genes within the common programs, aiming to identify potential biomarkers that could be targeted to inhibit LN metastasis in breast cancer. We extracted genes originated from the “Metastasis” module, assembled three extensive cohorts of breast cancer samples (TGCA, METABRIC, and SCAN‐B), and performed differential expression genes (DEGs) analysis of samples with and without LN metastasis. Among the three cohorts, we identified 25 common genes that were upregulated in the samples with LN metastasis (Figure 5D). We then conducted DEGs analysis and K‐M survival analysis based on these genes and ultimately screened out three potential biomarkers (*CRABP2*, *ERBB2*, and *GLO1*) of LN metastasis in breast cancer by applying the following filter criteria: upregulated in tumor samples, risky factor for survival, upregulated in cancer cells (Figure 5E–H; Figure S6B,C, Supporting Information). *CRABP2* is a member of the fatty acid binding protein (*FABP*) family, which exhibits binding affinity for all‐trans retinoic acid; previous studies have elucidated its critical function during breast cancer invasion and metastasis.^[^ ^35^, ^36^ ^]^ *ERBB2*, also known as *HER2*, is a well‐established negative prognostic indicator in breast cancer and serves as a therapeutic target for the monoclonal antibody trastuzumab in addition to other anti‐*HER2* agents.^[^ ^37^, ^38^, ^39^ ^]^ Given that the role of *GLO1* in breast cancer has not been extensively studied, we focused on its potential as a biomarker of LN metastasis in breast cancer. We first explored *GLO1* mRNA expression levels in tumor and normal tissues using pan‐cancer datasets, and the results showed that *GLO1* mRNA expression levels were significantly upregulated in most cancer types (Figure 5I). Additionally, *GLO1* was more highly expressed in all breast cancer subtypes than in normal tissues (Figure S6D, Supporting Information). Besides, we utilized TISCH database and found that comparatively to other types of cells, *GLO1* was mainly expressed in cancer cells (Figure 5J). Furthermore, ST data from four LN metastasis biopsies were collected to examine *GLO1* distribution at the spatial level, and the results were consistent (Figure 5K; Figure S6E, Supporting Information). Moreover, *GLO1* expression levels positively correlated with LN metastasis, age, and *Ki67* level (Figure 5L). In addition, we collected multiple datasets and performed K‐M survival analyses and found that higher *GLO1* expression levels were significantly associated with a more unfavorable prognosis in patients with breast cancer (Figure 5M; Figure S6F, Supporting Information). Taken together, our findings indicate that GLO1 is a promising biomarker of LN metastasis in breast cancer.

### 2.8 *GLO1* Depletion and Pharmacological Inhibition Hinder Proliferative and Metastatic Capacities of Breast Cancer Cells In Vitro

**Figure 6:**
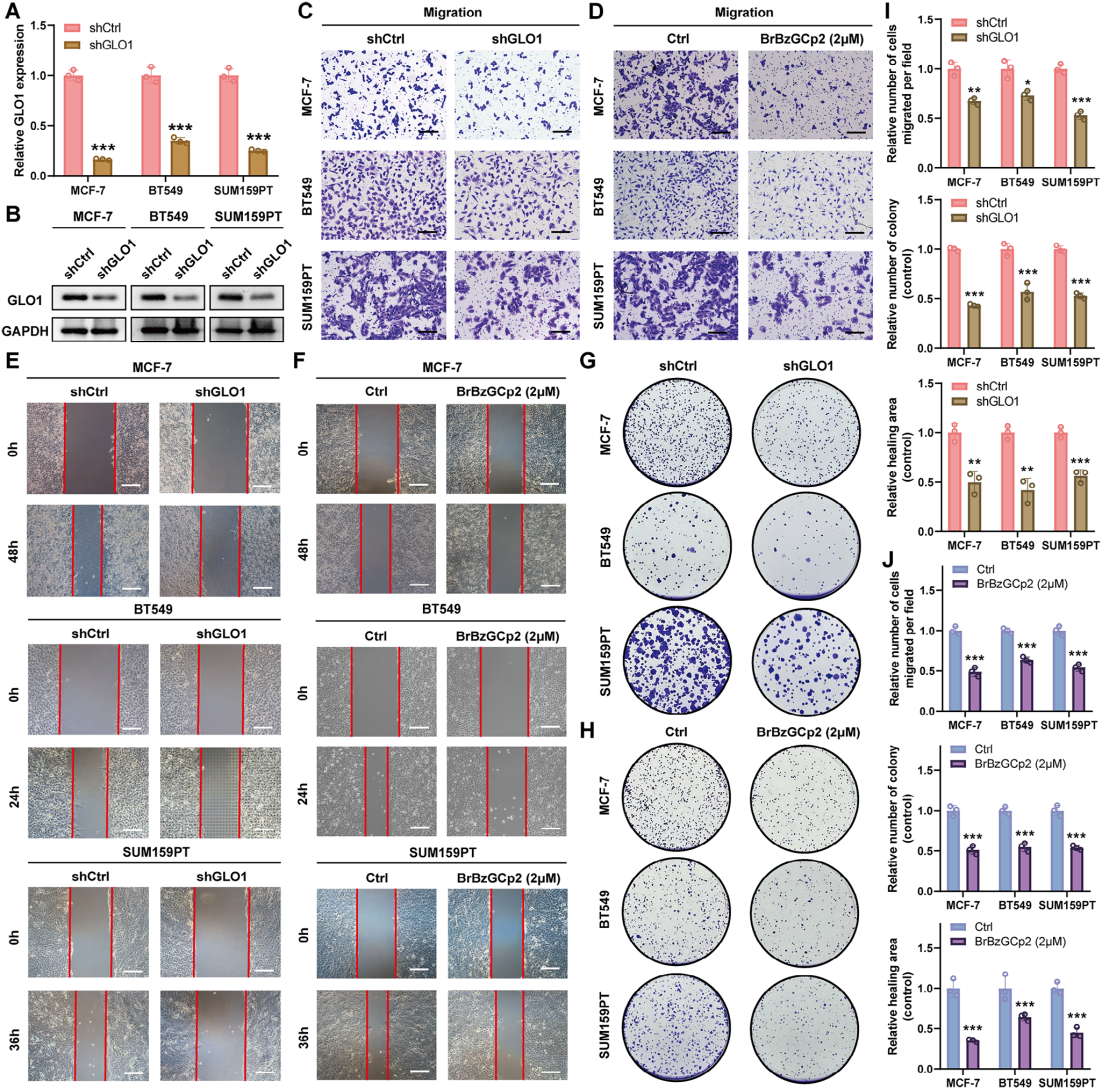
*GLO1* depletion and pharmacological inhibition hinder proliferative and metastatic capacities of breast cancer cells. A) qRT‐PCR confirmed the suppressive efficacy of *GLO1* mRNA expression. B) MCF‐7, BT549, and SUM159PT cells were stably transduced with lentivirus encoding short hairpin RNAs targeting *GLO1* (sh*GLO1*) or negative control RNA (shCtrl). Then, the cells were assayed for *GLO1* expression. C,D) Transwell assays detected the ability of breast cancer cells to migrate. E,F) Wound healing assays detected the ability of breast cancer cells to invasion. G,H) Colony assays detected the ability of breast cancer cells to proliferation. I,J) Histograms showing differences of number of cells migrated per filed, relative healing area, and relative number of colonies. (* means p < 0.05, ** means p < 0.01, and *** means p < 0.001).

We investigated the inhibitory effects of *GLO1* depletion on the metastatic capacity of breast cancer cells. A unique short hairpin RNA (shRNA) was used to effectively and stably suppression *GLO1* expression in breast cancer cells. The efficacy of this suppression was subsequently validated by qRT‐PCR and Western blotting analyses (**Figure** **6**A,B). Subsequently, we observed that the depletion of *GLO1* significantly inhibited cell proliferation, migration, and invasion, as demonstrated by transwell, colony formation, and wound healing assays (Figure 6C,E,G,I).

As inhibitors targeting *GLO1* have been developed,^[^ ^40^, ^41^ ^]^ we utilized a *GLO1* inhibitor (S‐p‐bromobenzyl glutathione cyclopentyl diester [*BrBzGCp2*]) to explore whether pharmacological inhibition of *GLO1* possesses a similar effect. Consistent with our hypothesis, we found that breast cancer cells treated with *BrBzGCp2* significantly reduced ability to proliferate and metastasize, as validated by Transwell, colony formation, and wound healing assays (Figure 6D,F,H,J). These findings confirmed that *GLO1* depletion and pharmacological inhibition hindered the proliferative and metastatic capacities of breast cancer cells in vitro.

### 2.9 *GLO1* Affects Lymphangiogenesis and LN Metastasis In Vitro and In Vivo

**Figure 7:**
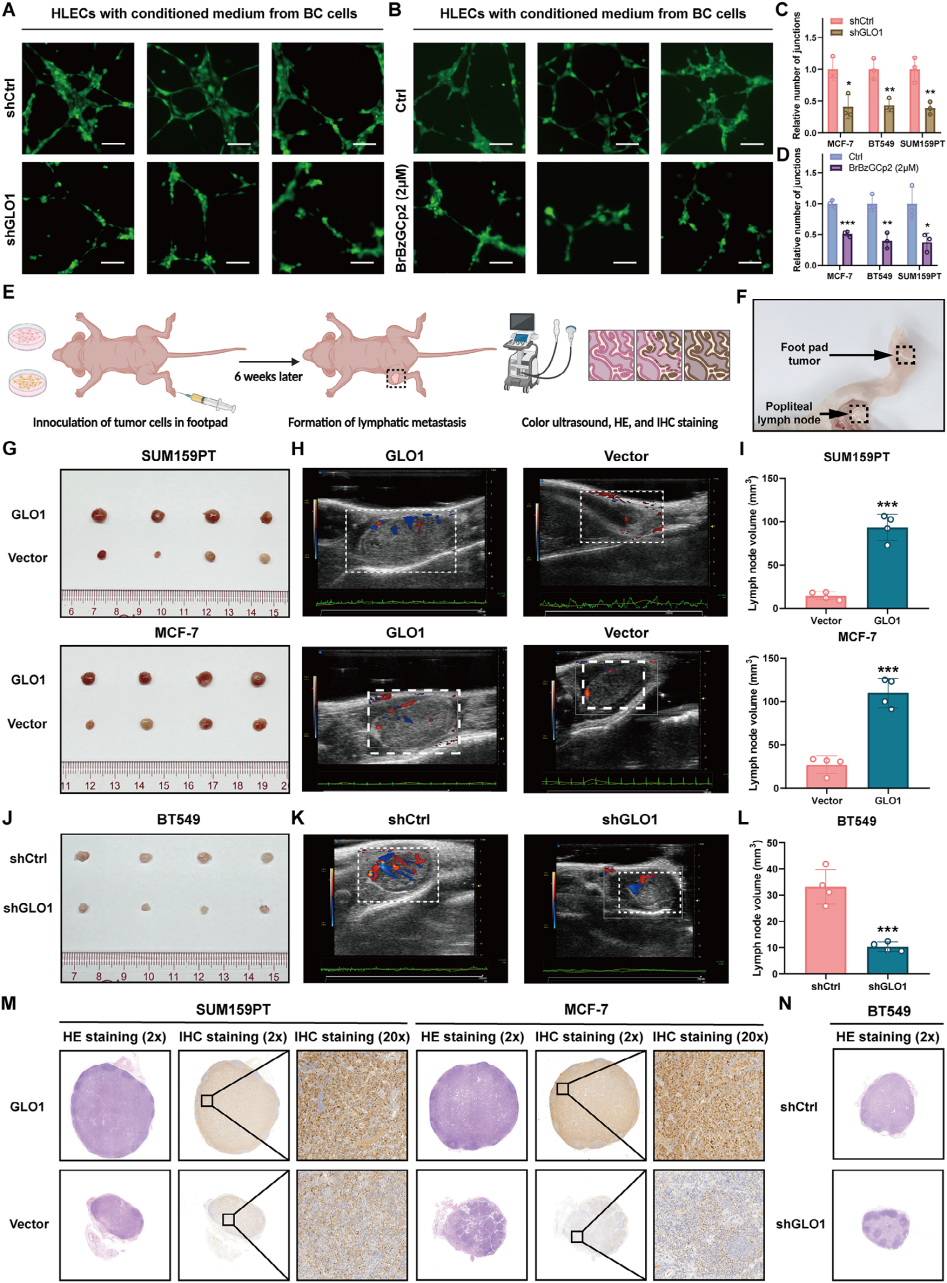
*GLO1* affects lymphangiogenesis and LN metastasis in vitro and in vivo. A,B) Tube formation assays using HLECs treated with conditioned medium from MCF‐7, BT549, and SUM159PT cells with different treatments. C,D) Histograms showing differences of relative number of junctions. E,F) Schematic representation for stablishing the nude mice model of popliteal LN metastasis (created with BioRender.com). G) Representative images of enucleated popliteal LNs for groups (n = 4). H) Representative images of popliteal LNs for groups using color ultrasound. I) Histogram showing differences of LN volume for groups. J) Representative images of enucleated popliteal LNs for groups (n = 4). K) Representative images of popliteal LNs for groups using color ultrasound. L) Histogram showing differences of LN volume for groups. M,N) HE and IHC staining of the mice metastatic LN tissues. (* means p < 0.05, ** means p < 0.01, and *** means p < 0.001).

Considering that angiogenesis and lymphangiogenesis are crucial for LN metastasis, the potential influence of *GLO1* on tube formation capability was evaluated.^[^ ^42^, ^43^ ^]^ Comparison of the sh*GLO1*‐conditioned medium with the control medium indicated a significant reduction in endothelial tube formation after exposure to the sh*GLO1*‐conditioned medium (**Figure** **7**A,C). Additionally, compared with the control‐conditioned medium, the *BrBzGCp2*‐conditioned medium caused a significant reduction in endothelial tube formation (Figure 7B,D). These results suggest that *GLO1* affects lymphangiogenesis and LN metastasis in vitro.

To investigate the role of *GLO1* in LN metastasis, an in vivo popliteal LN metastasis model was established. We injected SUM159PT and MCF‐7 breast cancer cells into the footpads of immunodeficient BALB/c nude mice and excised metastatic LNs from the established model after a period of six weeks (Figure 7E,F). We assessed the differences using color ultrasound, HE, and IHC staining. The LN volumes in the *GLO1* overexpression group were significantly greater than those in the vector control group (Figure 7G–I,M). In contrast, when BT549 breast cancer cells were injected into the footpads of BALB/c nude mice, LNs from the *GLO1* knockdown group presented noticeably smaller volumes (Figure 7J–L,N). Collectively, these findings corroborate that *GLO1* affects lymphangiogenesis and LN metastasis both in vitro and in vivo.

### 2.10 *GLO1* Promotes Lymphangiogenesis and LN Metastasis via a *VEGFA*‐Dependent Manner

**Figure 8:**
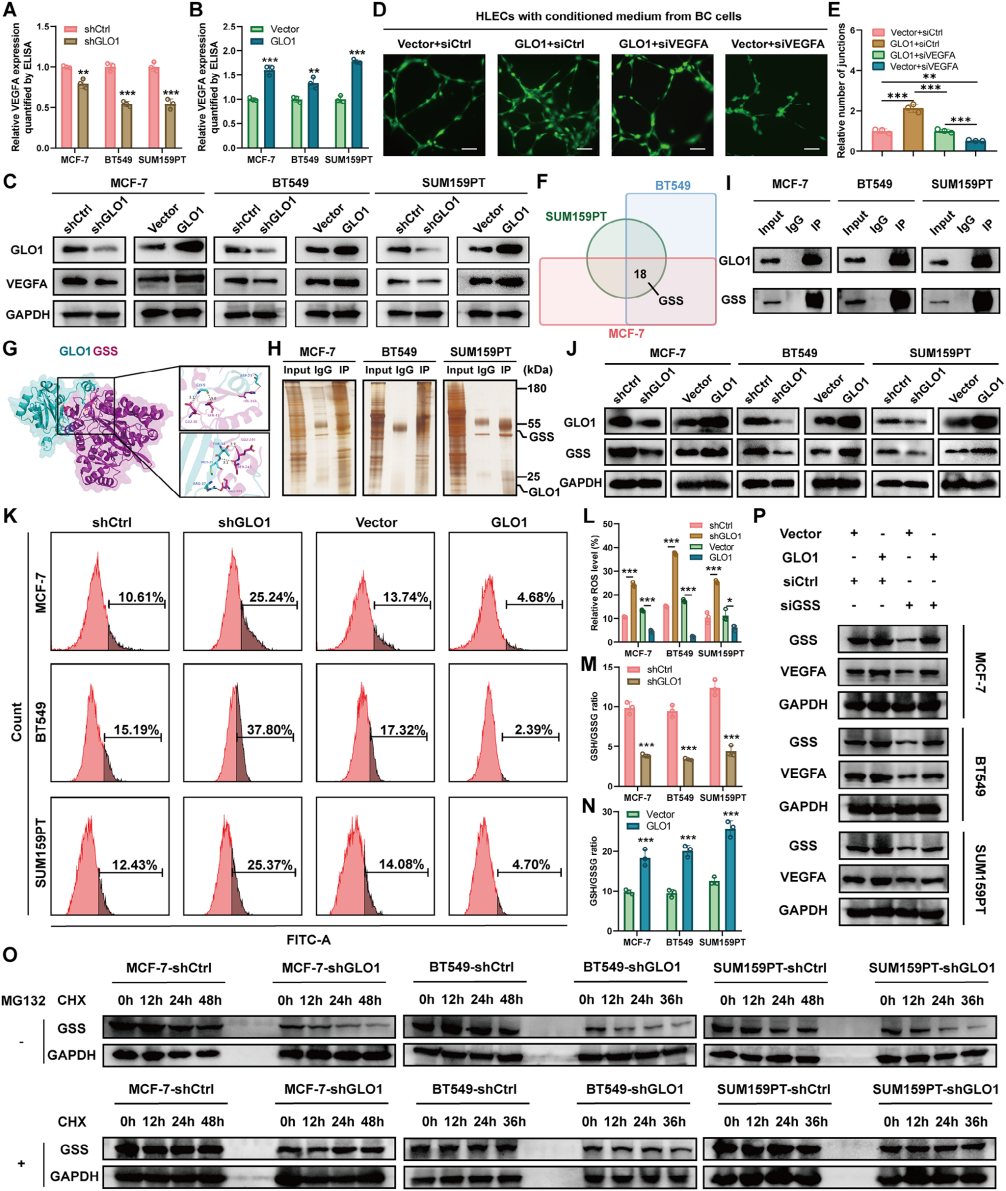
*GLO1* promotes lymphangiogenesis and LN metastasis via a *VEGFA*‐dependent manner and *GLO1* could interact with *GSS*. A) ELISA showing the effect of *GLO1* knockdown on *VEGFA* secretion. B) ELISA showing the effect of *GLO1* overexpression on *VEGFA* secretion. C) Western blotting showing the effect of *GLO1* knockdown and overexpression on *VEGFA* protein expression. D) Tube formation assays using HLECs treated with conditioned medium from BT549 cells with different treatments. E) Histograms showing differences of relative number of junctions. F) Venn plot showing the intersection proteins interacted with *GLO1* among three breast cancer cell lines using LC–MS/MS. G) Cartoon plots showing the structure and interaction between *GLO1* and *GSS*. H) Silver staining showing the differences of binding proteins among Input, IgG, and IP groups. I) Co‐IP assay confirmed the interaction between *GLO1* and *GSS*. J) Western blotting showing the effect of *GLO1* knockdown and overexpression on *GSS* protein expression. K) ROS levels were assessed via flow cytometry in breast cancer cell lines with different treatments. L) Histograms showing the relative ROS levels among different treatments. M,N) Histograms showing the GSH/GSSG ratios among different treatments. O) Western blotting showing the effect of *GLO1* knockdown on *GSS* stability in breast cancer cells incubated with CHX or MG132 at the indicated time points. P) *GSS* was inhibited in *GLO1*‐overexpressed breast cancer cells to determine whether the upregulated expression of *VEGFA* induced by *GLO1* overexpression could be rescued by *GSS* depletion. (ns means no significance, * means p < 0.05, ** means p < 0.01, and *** means p < 0.001).

Previous studies have documented the biological and clinical significance of *VEGF* family members (*VEGFA*, *VEGFB*, *VEGFC*, and *VEGFD*) in lymphangiogenesis and LN metastasis in various cancer types. Thus, we investigated whether *GLO1*‐mediated lymphangiogenesis was dependent on *VEGF* family members. We first verified the correlation between *GLO1* and the expression of each *VEGF* family gene using the bc‐GenExMiner database (https://bcgenex.ico.unicancer.fr/BC‐GEM) and found that only *VEGFA* was positively correlated with *GLO1* expression (Figure S7A, Supporting Information). Based on these results, we hypothesized that *GLO1* promotes lymphangiogenesis and LN metastasis via a *VEGFA*‐dependent manner. To validate this hypothesis, an enzyme‐linked immunosorbent assay (ELISA) assay was conducted to quantify the *VEGFA* levels in conditioned medium derived from breast cancer cells with either *GLO1* knockdown or overexpression. The results demonstrated a significant reduction in *VEGFA* expression in the conditioned medium from *GLO1* knockdown cells, whereas an obvious increase in *VEGFA* levels was observed in the medium from cells with *GLO1* overexpression (**Figure** **8**A,B). Western blotting analysis further showed that *GLO1* upregulated *VEGFA* expression at the protein level (Figure 8C). Next, we explored whether *VEGFA* is required for *GLO1*‐induced lymphangiogenesis and LN metastasis. We conducted tube formation assays and found that *VEGFA* downregulation rescued *GLO1*‐promoted lymphangiogenesis in vitro (Figure 8D,E). Collectively, we found that *GLO1* might promote lymphangiogenesis and LN metastasis via a *VEGFA*‐dependent manner.

### 2.11 *GLO1* Interacts with *GSS* and Maintains Homeostasis of Glutathione (*GSH*) and Reactive Oxygen Species (*ROS*) Levels

Elucidation of protein‐protein interactions is essential for understanding the molecular mechanisms underlying cellular processes.^[^ ^44^, ^45^ ^]^ We used liquid chromatography‐tandem mass spectrometry (LC–MS/MS) to identify proteins interacting with *GLO1* through immunoprecipitation (IP) of protein lysates from breast cancer cells. Eighteen common proteins were found in the LC–MS/MS results of the three breast cancer cell lines. Among these proteins, we observed *GSS*, a protein known to be associated with tumor progression (Figure 8F). *GSS* participates in *GSH* synthesis, and *GSH* serves a critical antioxidant function within cellular environments by neutralizing free radicals and ROS, thereby safeguarding cells against oxidative damage.^[^ ^46^, ^47^ ^]^ Overexpression of *GSH* has been documented in numerous tumor types, with substantial evidence supporting its resistance to various anticancer therapies.^[^ ^48^, ^49^, ^50^, ^51^ ^]^ We found that *GSS* was upregulated in most cancer types including breast cancer (Figure S7B, Supporting Information). In addition, we used molecular docking to predict the possibility of an interaction between the two proteins, indicating that *GLO1* is likely to interact with *GSH* (Figure 8G). Silver staining of the eluted protein fractions revealed significant *GSH* enrichment in the IP group, suggesting a potential interaction between *GLO1* and *GSH* (Figure 8H). Furthermore, a co‐immunoprecipitation (co‐IP) assay confirmed that endogenous *GSH* was enriched in the protein complex immunoprecipitated using the *GLO1* antibody in breast cancer cell lines (Figure 8I). Besides, we analyzed the association between *GLO1* and *GSH* mRNA expression levels. We found no obvious correlation between *GLO1* and *GSH* mRNA expression levels, indicating that they are more likely to interact at the protein level (Figure S7C,D, Supporting Information). Moreover, Western blotting further revealed that *GLO1* knockdown could downregulate *GSS* expression, whereas *GLO1* overexpression could upregulate *GSS* expression at the protein level (Figure 8J).

Subsequently, flow cytometry‐based detection of ROS was conducted and our findings demonstrated that *GLO1* depletion significantly elevated the production of cellular ROS levels, whereas *GLO1* overexpression resulted in excessive decrease of ROS levels (Figure 8K,L). In addition, we explored whether *GLO1* could maintain homeostasis of *GSH* and ROS levels. We performed *GSH* quantification and found that knockdown of *GLO1* significantly decreased the *GSH*/*GSSG* ratio, whereas *GLO1* overexpression enhanced the *GSH*/*GSSG* ratio (Figure 8M,N). Taken together, we verified that *GLO1* could interact with *GSS* and maintain the homeostasis of *GSH* and ROS levels.

### 2.12 *GLO1* Could Stabilize *GSS* and Prevent it from Proteasomal Degradation

**Figure 9:**
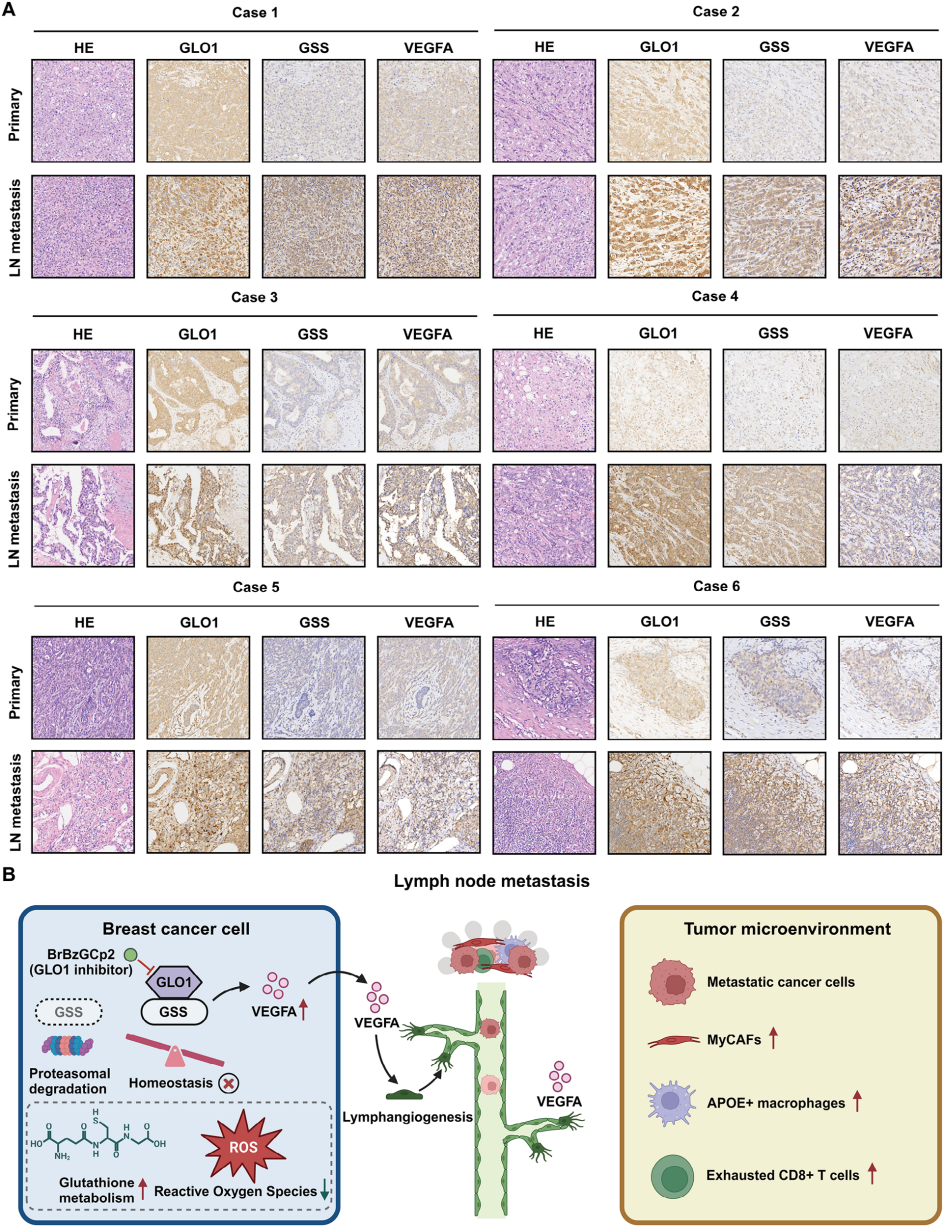
Clinical verification using IHC staining and schematic diagram of this study. A) Clinical verification of *GLO1*, *GSS*, and *VEGFA* protein expression levels using HE and IHC staining (n = 6). B) Schematic diagram describing that *GLO1* could promote lymphangiogenesis in breast cancer and cells such as myCAFs, *AOPE*+ macrophages, and exhausted *CD8*+ T cells were enriched and activate in breast cancer lymph node metastatic TME (created with BioRender.com).

The proteasome is crucial for the degradation of the majority of cellular proteins through a precisely regulated mechanism, thereby exerting control over numerous cellular processes such as the cell cycle, transcription, signaling, trafficking, and protein quality control.^[^ ^52^ ^]^ Proteasomal degradation plays a vital role in the maintenance of cellular and organismal homeostasis, and impairments or malfunctions in this process are linked to various human pathologies, including cancer.^[^ ^53^ ^]^ As *GSS* had been previously reported to be degraded by proteasomes in cells,^[^ ^54^ ^]^ we hypothesized that *GLO1* could stabilize *GSS* and prevent it from proteasomal degradation. Cycloheximide (CHX) is frequently employed in proteasomal degradation research because it non‐selectively inhibits protein biosynthesis within cells.^[^ ^55^ ^]^ Following treatment with CHX, *GLO1* knockdown accelerated the degradation of *GSS* in breast cancer cell lines, and this effect was reversed when MG132 (a proteasome inhibitor) was added to the breast cancer cell lines (Figure 8O). These results confirmed that *GLO1* could stabilize *GSS* and prevent its proteasomal degradation. Besides, we conducted Western blotting and found that the interaction between *GLO1* and *GSS* could affect *VEGFA* expression at protein level, that is, the upregulated expression of *VEGFA* induced by *GLO1* overexpression could be rescued by *GSS* depletion (Figure 8P). Moreover, breast cancer tissues comprising primary lesions and their corresponding LN metastatic tissues were collected. We conducted IHC staining and found that *GLO1*, *GSS*, and *VEGFA* were consistently upregulated in LN metastatic tissues compared to primary sites (**Figure** **9**A).

## 3 Discussion

Generally, patients diagnosed with breast cancer have a poor prognosis, particularly when LN metastasis is present, as this condition markedly diminishes patient survival rates. Most studies have focused on distant organ metastases in breast cancer. However, limited research has been conducted on the specific mechanisms underlying LN metastasis. Meanwhile, a barrier persists in research concerning the remodeling of the LN microenvironment by tumor cells. Consequently, we performed detailed analyses of the molecular mechanisms underlying LN metastasis in breast cancer using multi‐omics approaches. We investigated the regulatory mechanisms of the TME within the LNs that might influence cancer cell metastasis and colonization. Our study placed particular emphasis on identifying the differences between LN+ and LN‐ tissues through scRNA‐seq. In our analysis of 28 LN samples, we identified ten major cell types. Furthermore, the subclusters within each major cell type demonstrated diverse characteristics. Due to the heterogeneity of tissues, marker genes for CAFs differ across various cancers, leading to their classification into different subtypes with unique functions.^[^ ^56^, ^57^ ^]^ We found that myCAFs enrichment might promote LN metastasis in breast cancer. MyCAFs aid in tumor growth and spread by preventing lymphocyte infiltration and altering the tumor's extracellular matrix of tumors in numerous cancer types.^[^ ^58^, ^59^ ^]^ Besides, a previous clinical trial showed that myCAFs enrichment was related to primary resistance to ICI therapy.^[^ ^60^ ^]^ Revealing these immune‐evasive tumors to the immune system is crucial for eliminating cancer cells. Researchers have explored the application of antibodies or inhibitors to target the activation, function, and normalization of CAFs in several ongoing clinical trials.^[^ ^61^ ^]^ Given that genes highly expressed by CAFs are also crucial for normal tissues, their efficacy and potential side effects must be carefully monitored. We also observed that the TME might be related to the interplay between *APOE*+ macrophages and exhausted *CD8*+ T cells in LN metastasis in breast cancer and that *APOE*+ macrophages displayed M2‐like macrophage characteristics associated with reduced antitumor activity, promotion of tumor angiogenesis, and enhanced tumor progression.^[^ ^62^, ^63^ ^]^ Previous studies showed that the interplay between *C1Q*+ macrophages (highly expressed *APOE*) and exhausted T cells led to the dysfunctional immune circuit in clear cell renal cell carcinoma, lung cancer, cervical cancer, etc.^[^ ^64^ ^]^ In addition, the combination of *APOE* inhibitors and immune checkpoint inhibitors has shown promising prospects, indicating their potential application in cancer therapy.^[^ ^33^ ^]^

In the present study, we identified five common expression programs characterized by diverse biological functions and cellular states, including proliferation, metastasis, OXPHOS, hypoxia, and interferon. Among these hub genes, we screened out *GLO1* as a promising biomarker for LN metastasis in breast cancer. Besides, through the integration of multi‐omics bioinformatics analyses, in vitro and in vivo experiments, we confirmed that *GLO1* could facilitate LN metastasis. *GLO1*, located on chromosome 6p21.2,^[^ ^65^ ^]^ is a crucial enzyme that controls methylglyoxal levels in cells; its expression is inversely related to the production of methylglyoxal, a byproduct of glycolysis that exhibits cytotoxic properties and inhibits cellular proliferation.^[^ ^66^, ^67^ ^]^ *GLO1* is markedly overexpressed in most tumors, and its elevated expression is associated with an unfavorable prognosis.^[^ ^68^, ^69^, ^70^, ^71^ ^]^ Additionally, *GLO1* overexpression leads to resistance against multiple drugs in cancer.^[^ ^72^ ^]^ Moreover, a recent investigation revealed a crucial role for ECs‐originated *GLO1* in peritumor electroacupuncture, emphasizing that targeting *GLO1* could be a promising strategy for improving vascular normalization and TNBC treatment outcomes.^[^ ^73^ ^]^ However, we found that *GLO1* was consistently upregulated in all breast cancer subtypes and predominantly expressed in malignant cancer cells. Besides, the role of *GLO1* in mediating the invasion and metastasis of breast cancer remains unclear.

Our results showed that *GLO1* depletion and pharmacological inhibition could hinder proliferative and metastatic capacities of breast cancer cells. In addition, *GLO1* could promote lymphangiogenesis and LN metastasis via *VEGFA*‐dependent manner. Mechanistically, *GLO1* could interact with *GSS* and inhibit its proteasomal degradation, thereby maintaining *GSH* homeostasis as well as *ROS* levels, and preventing tumor cells elimination. Moreover, the upregulation of *VEGFA* induced by *GLO1* overexpression could be rescued by *GSS* depletion. We hypothesized that *GSH* protects cells during lymphangiogenesis by modulating ROS levels. *GSH* mitigates oxidative stress under excessive ROS conditions, supporting normal cellular function and maintaining the microenvironment, thereby facilitating the healthy progression of lymphangiogenesis. Once the aforementioned alterations occur, the TME status is transformed into a conducive “soil” that promotes lymphangiogenesis as well as lymphatic metastasis in breast cancer (Figure 9B).

Although *GLO1* is a promising target, its clinical transformation still needs to be considered. The primary therapeutic application of *GLO1* inhibitors is cancer chemotherapy, particularly for tumors exhibiting high *GLO1* expression. Additionally, *GLO1* inhibitors have been used as adjunct therapies in cancer chemotherapy to counteract multidrug resistance.^[^ ^72^ ^]^ Clinical evaluations have shown promising results in targeting glioblastoma multiforme and breast cancer, indicating its capacity for anti‐tumor therapy.^[^ ^74^ ^]^ Besides, our in vitro experiments confirmed their potential use in cancer therapy. However, given that high glycolytic rate and elevated *GLO1* expression are pivotal elements in its therapeutic potential, these aspects should be carefully evaluated. Besides, while initial studies with proliferating lymphocytes in vitro and tumor‐bearing mice suggested that adverse effects are limited,^[^ ^75^ ^]^ normal‐tissue toxicity or compensatory mechanisms should be considered when proposing *GLO1* inhibitors for cancer therapy since *GLO1* is broadly relevant to cellular metabolism. In addition, the feasibility of combining *GLO1* inhibitors with angiogenesis inhibitors or other targeted (immune checkpoints and *CDK4/6*) agents, warrants further investigation.^[^ ^76^ ^]^

Notably, our study has certain limitations. First, our findings were predominantly derived from extensive big‐data analyses and the current sample size might not be representative of broad conclusions. Consequently, in‐house cohorts need to be collected and prospective validations as well as additional experiments employing advanced technologies, such as metabolomics, are essential to make it reliable for larger, more diverse populations, which would help in validating the generalizability of our study findings. Second, more detailed mechanisms are necessary to elucidate the protein‐protein interactions between *GLO1* and *GSS*, the underlying *VEGFA*‐dependent mechanisms, as well as the crosstalk between *GLO1* activity and TME components. Third, our study did not extensively discuss the subtypes of samples that could influence the TME's composition and metastatic mechanisms since different subtypes might lead to varying immune infiltration patterns and cell‐type distributions. Incorporating subtype information is imperative to improve the clinical translation of the findings and potentially guide personalized therapeutic approaches targeting *GLO1* or other subtype‐specific pathways. Furthermore, given that metastasis is a multifaceted process encompassing various stages, including in situ tumor growth, angiogenesis, EMT, invasion, infiltration, circulation survival, extravasation, dormancy, metastatic tumor growth, and interaction with TME components, it is necessary to elucidate the intricate mechanisms by which *GLO1* influences the progression of LN metastasis in breast cancer.^[^ ^77^ ^]^

## 4 Conclusion

Collectively, our study offers novel perspectives on the microenvironment remodeling of LN metastasis in breast cancer, suggesting that *GLO1* might be a key target for LN metastasis in breast cancer.

## 5 Experimental Section

### Ethics Statement

Sun Yat‐sen University Cancer Center's Institute Research Ethics Committee approved this study. All animal experiments were conducted in accordance with protocols approved by the Sun Yat‐sen University Cancer Center Animal Care and Use Committee (approval ID: L102012024080E). The tumor tissues utilized for CAFs extraction, IHC, and mIF staining in breast cancer patients were obtained retrospectively from Sun Yat‐sen University Cancer Center (approval ID: SL‐B2024‐637‐01) and Guangdong Provincial People's Hospital (approval ID: KY2024‐691‐01).

### Data Collection

Patients according with the following criteria were included: (1) female. (2) confirmed diagnosis of metastatic breast cancer by postoperative histopathology. (3) lesions located in the axillary LNs. A total of 28 LN samples (23 with metastases and 5 with non‐metastases) were finally collected from 23 patients, including 6 samples from GSE161529 (6 with metastases),^[^ ^78^ ^]^ 8 samples from GSE167036 (8 with metastases),^[^ ^79^ ^]^ 10 samples from GSE180286 (5 with metastases and 5 with non‐metastases),^[^ ^80^ ^]^ and 4 samples from GSE225600 (4 with metastases).^[^ ^81^ ^]^ Written informed consent was procured from all study participants. Table S1 (Supporting Information) displays the detailed clinical features of all the samples included in this study.

### Quality Control and Batch Correction of scRNA‐seq

Cell quality control was conducted utilizing the Seurat R package.^[^ ^82^ ^]^ Cells were defined as low‐quality if they met the following criteria: nFeature_RNA<500, nFeature_RNA>7500, mitochondrial genes>50%, and erythrocyte genes>10%. Doublet elimination was conducted through “DoubletFinder” R package (https://github.com/chris‐mcginnis‐ucsf/DoubletFinder) and manual deletion when the cell population simultaneously overexpresses two or more classic cell type markers.^[^ ^83^ ^]^ It utilized the “harmony” R package (https://github.com/immunogenomics/Harmony) to eliminate batch effects from sequencing data obtained from various samples.

### Cell Clustering and Annotation

Cells clustering was predominantly accomplished by “RunTSNE”, “RunUMAP” function, “FindNeighbors” function, and “FindClusters” function within the “Seurat” R package. The “FindAllMarkers” function was utilized to identify genes demonstrating specific expression patterns within individual cell clusters.

### CNV Analysis

It utilized CNV analysis through the inferCNVpy (https://github.com/icbi‐lab/infercnvpy).^[^ ^84^ ^]^ inferCNVpy was a computational tool designed for detecting CNVs. It operates by comparing gene expression profiles of tumor cells against a defined reference population to identify CNV deviations. Genes were organized by chromosome and genome location, and average gene expression in genomic regions was compared with the reference cells. Chromosome heatmap was drawn after running leiden clustering.

### Trajectory and Cellular Communication Analysis

The Monocle 2 algorithm was utilized to conduct pseudo‐time trajectory analysis in order to map subtype differentiation and conversion.^[^ ^85^ ^]^ Additionally, DDRTree was employed for dimension‐reduction analysis. Through CellChat R package, it were able to predict potential cell‐to‐cell communication between stromal/immune cells and malignant cells.^[^ ^86^ ^]^

### Identification of Shared Expression Modules

Utilizing the consensus non‐negative matrix factorization algorithm (cNMF; https://github.com/dylkot/cNMF), transcriptional modules were identified as meta‐signatures and their corresponding scores were computed. Subsequently, a meta‐signature was constructed and the samples were subjected to hierarchical clustering.

### Patient‐Derived CAFs Extraction

Breast cancer tissues were obtained from independent patients with invasive ductal carcinoma after surgical excision. Tissues were cut into pieces and digested using hyaluronidase (150 units mL^−1^, Sigma) and collagenase type I (1 mg mL^−1^, Sigma) at 37 °C with agitation for 8 h in DMEM. The digested tissues were incubated at room temperature for 5 min and the supernatant was harvested to new tubes and centrifuged at 300 g for 3 min. The supernatant was discarded and the CAFs were resuspended in DMEM/F12 with 10% Fetal Bovine Serum (FBS). The cells were cultured at 37 °C with 5% CO2. The fibroblasts were expended and passaged in 15 cm dishes. Fibroblasts up to 5 passages were used for further experiments to diminish the clonal selection and bias.^[^ ^87^, ^88^ ^]^ CAFs were validated by immunofluorescence for *α‐SMA* (14395‐1‐AP).

### Cell Lines and Culture Conditions

Breast cancer cell lines (MCF‐7, BT549, and SUM159PT) were acquired from the American Type Culture Collection and HLEC was purchased from Jennio Biotech Co.,Ltd. These cells were cultured according to a standardized protocol at a constant temperature of 37 °C in the absence of antibiotics.

### Transient Transfection and Stable Cell Lines Construction

For siRNA‐mediated RNA interference, siRNAs specifically targeting *ACTA2* and *VEGFA*, as well as non‐targeting control, were synthesized and procured from GenePharma (Suzhou, China). The siRNAs were transfected into the cells using Lipofectamine 3000 (Invitrogen), following the protocol provided by the manufacturer. Stable knockdown and overexpression cell lines in breast cancer cell lines (MCF‐7, BT549, and SUM159PT) were achieved by infecting with lentivirus and screening with puromycin following standard procedures. Tables S2 and S3 (Supporting Information) presents the sequences of the siRNA and shRNA that were transfected into the cells, respectively.

### RNA Isolation and qRT‐PCR Analysis

Total RNA was extracted from cells using the RNA‐Quick Purification Kit (ES‐RN001, Shanghai Yishan Biotechnology Co.). Table S5 (Supporting Information) shows the primer sequences. qRT‐PCR was performed to determine the RNA levels on a Bio‐Rad CFX96 using the SYBR Green method (RR420A; Takara). The qRT‐PCR plates were purchased from NEST (402301; Wuxi NEST Biotechnology Co.). Comparative Ct method was used to normalize the RNA levels against *β*‐actin RNA.

### Western Blotting and Co‐IP Assay

For western blotting, cell lysates from MCF‐7, BT549, SUM159PT, and CAFs were prepared using RIPA lysis buffer to extract proteins, which were then separated by SDS‐PAGE (Beyotime) and transferred onto PVDF membranes (Millipore). Following 1 hour of blocking in nonfat milk, membranes were incubated with a primary antibody overnight at 4 °C and subsequent incubation with secondary antibodies at room temperature for 1 hour. For Co‐IP, protein extractions from MCF‐7, BT549, SUM159PT cell lines were conducted using IP lysis buffer. Each IP condition utilized 80% of the total protein lysate, with the remaining 20% designated as input. The IP samples, in conjunction with primary antibodies, were allowed to incubate at 4 °C overnight. Following this incubation period, protein A/G magnetic beads (HY‐K0202, MCE) were introduced and incubated at 4 °C for 2 h, followed by five washes. The beads were subsequently eluted in 1X SDS‐PAGE. Antibodies targeting *ACTA2* (14395‐1‐AP), *GLO1* (15140‐1‐AP), *VEGFA* (19003‐1‐AP), *GSS* (15712‐1‐AP), and *GAPDH* (60004‐1‐Ig) were applied. Blots were visualized using Immobilon Western Chemiluminescent HRP Substrate (Beyotime).

### Transwell Assay

MCF‐7, BT549, and SUM159PT cell lines were digested and reassembled. The experimental cells were introduced into the upper chambers in a medium devoid of fetal bovine serum (FBS), while a medium containing 20% FBS was introduced into the lower cross‐pore chambers. Following fixation with methanol, staining with crystal violet (0.1%), and imaging, all migrated cells were analyzed.

### HLECs Tube Formation Assay

The conditioned medium was harvested from cultured groups of breast cancer cells and CAFs. HLECs were cultured in 24‐well plates pre‐coated with Matrigel in the presence of conditioned medium. After a period of 6 h, the capillary‐like structures produced by HLECs were stained with Calcein‐AM and observed using a fluorescence microscope.

### Colony Formation Assay

MCF‐7, BT549, and SUM159PT cell lines with different treatments were cultured in 6‐well plates. Following a 14‐day incubation period, the colonies were fixed in methanol and subsequently stained with crystal violet (0.1%). The colonies within each well were then visualized and quantified.

### Wound Healing Assay

MCF‐7, BT549, and SUM159PT cell lines were cultured in 6‐well plates and transfected. Subsequent to the wound induction procedure, images of the wounds were captured under a microscope, and the ImageJ software was utilized to quantify the wound area and assess cell migration.

### Multiplex Immunofluorescent Staining

According to established protocols, paraffin‐embedded human tissue sections were employed for immunofluorescent staining. Sections were dewaxed in xylene, rehydrated with graded ethanol, had endogenous peroxidase activity blocked, and underwent antigen retrieval at elevated temperatures. Subsequently, the sections were permeabilized in TBST (PBS with 0.5% Triton X‐100) and incubated over‐night at 4 °C with the primary antibodies. Antibodies targeting *PanCK* (AF20164), *α‐SMA* (BM0002), *CD3* (AB16669), *CD20* (AB64088), *CD68* (DF7518), *CD8A* (AF20211), *PD‐1* (AF20083), *APOE* (AF300571) were applied. Primary antibodies were given in order, and then the secondary antibody and fluorophore were used for incubation.

### IHC Staining

Human and mouse tissue sections underwent deparaffinization using xylene followed by rehydration with a series of ethanol concentrations (100%, 95%, 85%, and 75%). Antigen retrieval was conducted before overnight incubation with the primary antibody at 4 °C to inhibit endogenous peroxidase activity. Subsequently, the sections were incubated for 20 minutes with an HRP‐conjugated secondary antibody at room temperature, and staining was carried out using diaminobenzidine (DAB) substrate (Dako). Following DAB treatment, the sections were counterstained with hematoxylin.

### ELISA Kit Assay

Cell supernatants were collected to quantify the secretion of *VEGFA* via ELISA kit assay (ELK Biotechnology, China) according to the guidelines provided by the manufacturer.

### Animal Experiments

Female BALB/c nude mice aged 4 weeks were purchased from Zhuhai BesTest Bio‐ Tech Co. Ltd. and housed at the Animal Facility of SYSUCC under controlled conditions. For the popliteal LN metastasis model, MCF‐7, BT549, and SUM159PT cell lines suspended in PBS were administered via injection into the footpads of BALB/c nude mice that were subsequently randomized into groups (n = 4 for each group). With 6 weeks post‐injection, the mice were humanely euthanized, and the popliteal LNs were surgically removed for analysis.

### GSH Quantification and Flow Cytometry‐Based Detection of ROS

Cellular *GSH* levels were quantified using the *GSH*/*GSSG* Quantification Kit (Beyotime). MCF‐7, BT549, and SUM159PT cell lines were treated with a specified compound for 48 h, followed by rinsing in chilled PBS three times. Subsequently, *GSH* levels were measured in accordance with the manufacturer's instructions. ROS levels were assessed using the DCFH‐DA probe from the Reactive Oxygen Species Assay Kit (Beyotime). Prior to incubation with the DCFH‐DA probe for 0.5 h, breast cancer cells were pre‐treated with specified agents for 48 h. Subsequently, the cells were harvested, washed in chilled PBS three times, and ROS levels were quantified using flow cytometry.

### Statistical Analysis

Statistical analyses within this research were executed using R, Python, and GraphPad Prism software. For comparison between two groups, Student's t‐test and Wilcoxon rank‐sum test were applied for normally and non‐normally distributed variables, respectively. One‐way ANOVA and Kruskal‐Wallis test was used for parametric non‐parametric multigroup comparisons, respectively. The results were presented as means ± standard deviations. Survival analysis was conducted using the “survival” and “survminer” R packages. The best cutoff value in each cohort was adopted to draw the K–M plot using the log‐rank test. Correlation analysis was performed using Spearman's method. All p values were two‐tailed and were reported in the figures and figure legends. p < 0.05 was considered statistically significant.

## Conflict of Interest

The authors declare no conflict of interest.

## Author Contributions

J.X., W.L., X.D., and H.W. contributed equally to this work. S.Z., W.X., and R.S. performed research design. J.X., W.L., X.D., and H.W. performed data collection. J.X., W.L., X.D., H.W., and X.O. performed data analysis. J.X., W.L., X.D., H.W., X.O., X.A., M.‐Y.S., A.Y., C.P., R.H., Y.X., H.T., Y.C., and J.‐Y.L. prepare manuscript. S.Z., W.X., and R.S. edited the manuscript. All authors contributed to the article and approved the submitted version.

## Supporting information



Supporting Information

Supporting Information

## Data Availability

Raw sequencing data for this study are available through the NCBI Gene Expression Omnibus (GEO) under accession number “GSE161529”, “GSE167036”, “GSE180286”, and “GSE225600”. Detailed information for each dataset has been listed in the Supplementary Files. Processed data for this study are available from the corresponding author upon reasonable request.
