# Supplementary material for: Paracrine Orchestration of Tumor Microenvironment Remodeling Induced by GLO1 Potentiates Lymph Node Metastasis in Breast Cancer
Source: Adv Sci (Weinh). 2025 Jun 10;12(32):e00722. doi: 10.1002/advs.202500722 (PMC12407353; doi:10.1002/advs.202500722)

ADVANCED SCIENCE Open Access

Supporting Information

for *Adv. Sci.*, DOI 10.1002/advs.202500722

Paracrine Orchestration of Tumor Microenvironment Remodeling Induced by GLO1 Potentiates Lymph Node Metastasis in Breast Cancer

Jindong Xie, Wenjian Liu, Xinpei Deng, Huan Wang, Xueqi Ou, Xin An, Min-Yi Situ, Anli Yang, Chuan Peng, Rongfang He, Yi Xie, Hailin Tang, Yuman Chen, Jie-Ying Liang, Ruonan Shao, Weikai Xiao* and Shaoquan Zheng**

Supplementary Figure Legends

Supplementary Figure S1. Single-cell atlas of LN metastasis in breast cancer. (A) UMAP plots showing the leiden clustering. **(B)** Heatmap showing CNV profiles inferred by scRNA-seq. **(C)** t-SNE plot of single cells profiled in the present study before and after batch correction by "harmony" R package (splited by dataset). **(D)** t-SNE plot of single cells profiled in the present study colored by datasets and LN status. **(E)** Feature plots for the canonical marker genes of cancer cells (*KRT8*), B cells (*CD79B*), myeloid cells (*LYZ*), mural cells (*TAGLN*), T cells (*TRBC2*), endothelial cells (*VWF*), NK cells (*XCL1*), CAFs (*LUM*), plasma cells (*MZB1*), and proliferation cells (*TOP2A*).

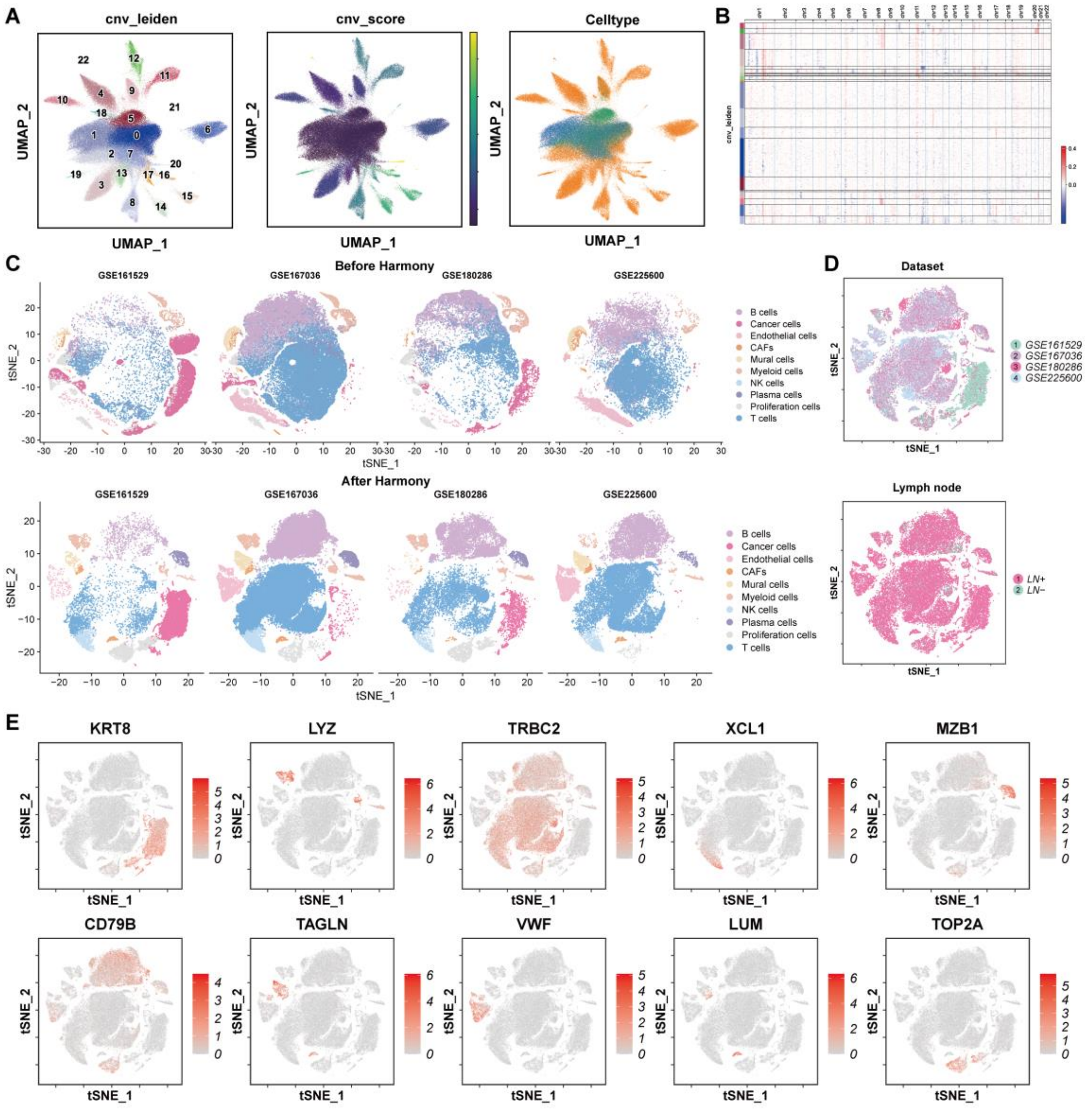

Supplementary Figure S2. Cell clustering and categorization of stromal cells in breast cancer LN tissues. (A) t-SNE plot and feature plot of the CAFs cells landscape. **(B)** t-SNE plot and feature plot of the ECs cells landscape. **(C)** t-SNE plot and feature plot of the mural cells landscape. **(D)** Pathway enrichment analyses of apCAFs, iCAFs, meCAFs, and SMCs using Gene Ontology (GO) database. **(E)** Ligands and receptors for signal communication between cancer cells and stromal cell subclusters. **(F)** Violin plots showing the different levels of *COL1A1*, *COL1A2*, *COL6A1*, *COL6A2*, and *MDK* between LN- and LN+ tissues in each stromal cell subcluster.

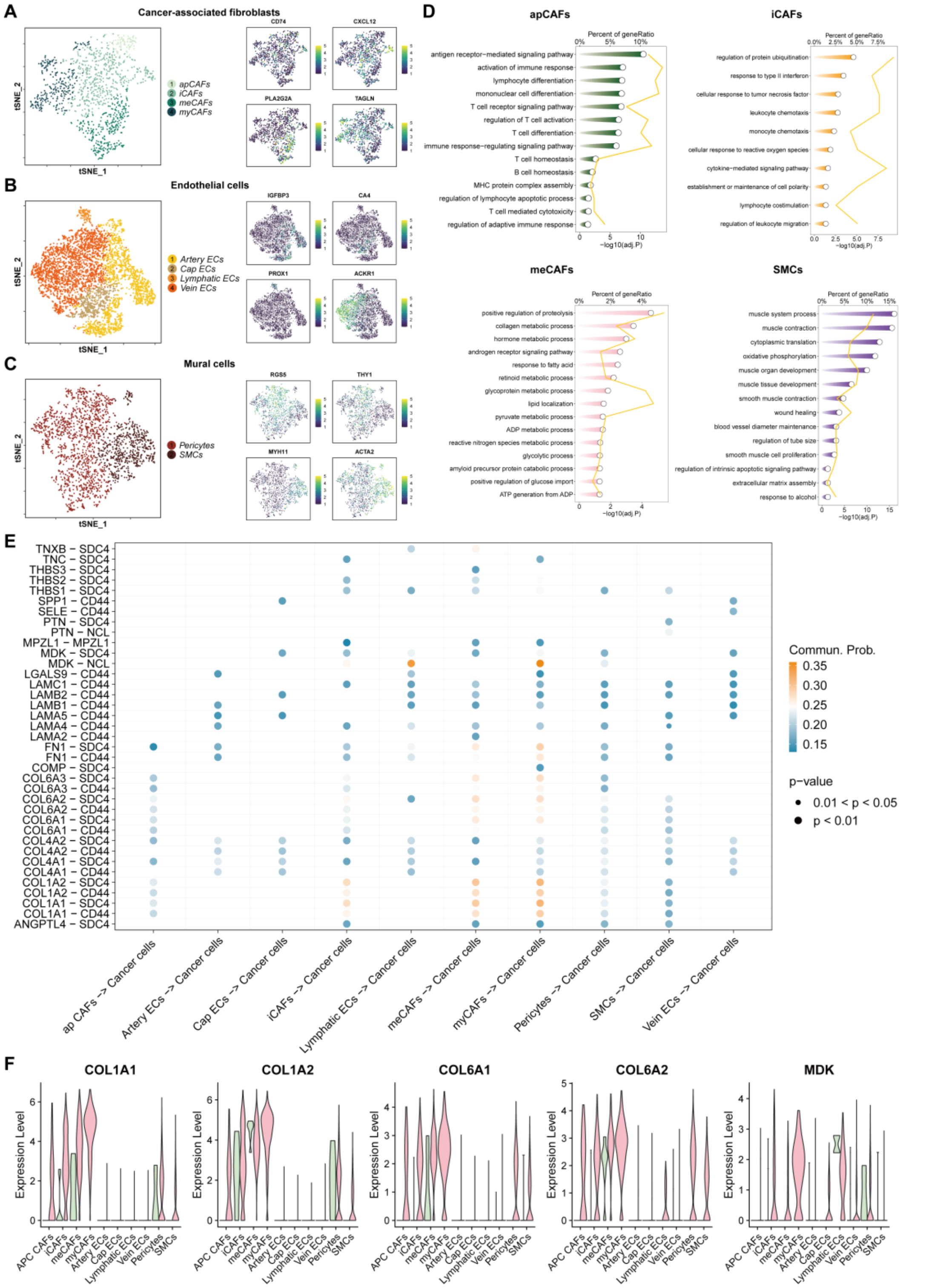

Supplementary Figure S3. Characterization of immunosuppressive myeloid cells in breast cancer LN tissues. (A) Feature plots showing the normalized expression of highly expressed genes in each myeloid cell subcluster. **(B)** Bar plot for cell proportion of myeloid cell clusters between LN- and LN+ tissues. **(C)** Dot plots showing the metabolic pathways activities among myeloid cell subclusters. **(D)** Cellchat analyses showing number of interactions among different cell types. **(E)** Heatmap showing the number of interactions among different cell types. **(F)** Ligands and receptors for signal communication between myeloid cell subclusters and cancer cells. **(G)** Violin plots showing the expression levels of *CD99*, *LGALS9*, *FN1*, and *SPP1* among myeloid cell subclusters.

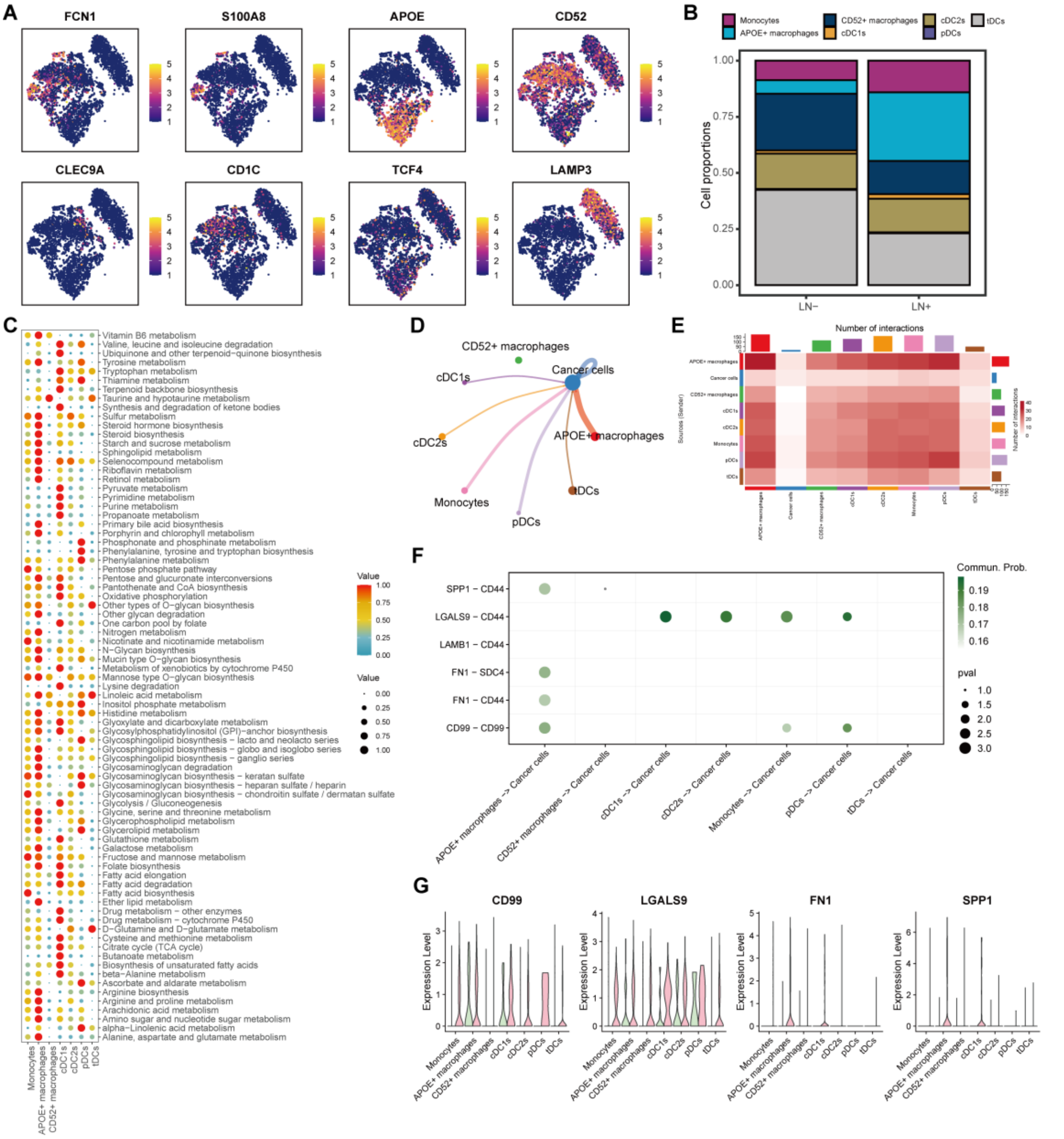

Supplementary Figure S4. T/NK cells were distinguished in breast cancer LN tissues. (A) Dot plots showing the expression levels of classic markers in each *CD4+* and *CD8+* T cell subcluster. **(B)** t-SNE plot of the Treg cells landscape. **(C)** Feature plots showing the normalized expression of highly expressed genes in each Treg cell subcluster. **(D)** Bar plot for cell proportion of Treg cell clusters between LN- and LN+ tissues. **(E)** Scatter plots for the different percentage of each Treg subcluster between LN- and LN+ tissues. **(F)** Heatmap showing the scaled expression levels of co-stimulators, co-inhibitors, and T-function markers among different breast cancer subtypes. **(G)** Heatmap showing the activated pathways and biological processes among *CD4+* and *CD8+* T cell subclusters using hallmark gene sets. **(H)** Scatter plots showing the correlation between *APOE+* macrophages score and *CD8+*Tex score. **(I)** HE staining of each sample. **(J)** Heatmap showing the importance of *APOE+* macrophages and *CD8+* T cell subclusters during *MHC-I* and *CD86* signaling pathways network. **(K)** Violin plots showing the expression levels of certain genes in *MHC-I* and *CD86* signaling pathways among *APOE+* macrophages and *CD8+* T cell subclusters.

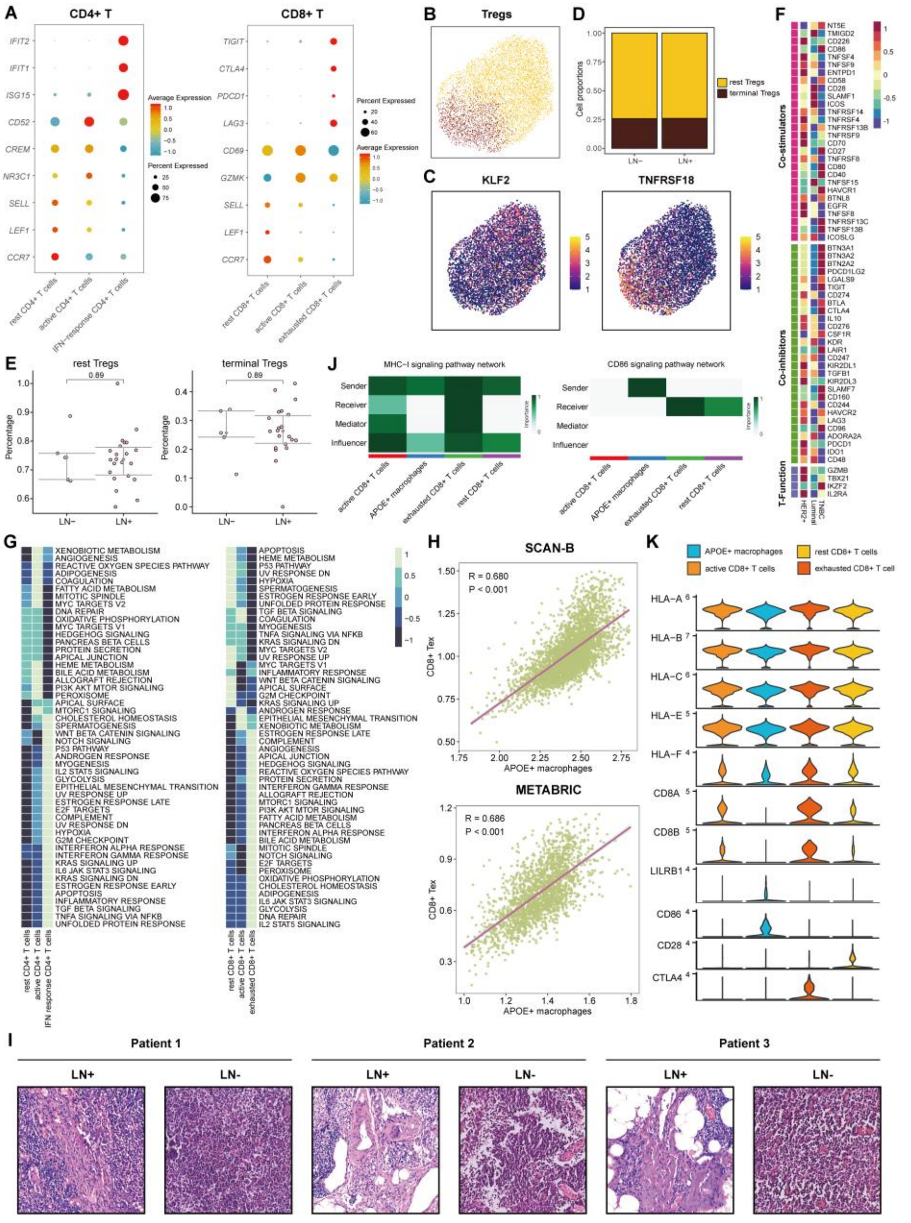

Supplementary Figure S5. Transcriptomic diversity of B and plasma cells in breast cancer LN tissues. (A) t-SNE plot of the B/Plasma cells landscape. **(B)** Feature plots showing the normalized expression of highly expressed genes in each B/Plasma cell subcluster. **(C)** Bar plot for cell proportion of B/Plasma cell clusters between LN- and LN+ tissues. **(D)** Scatter plots for the different percentage of each B/Plasma cell subcluster between LN- and LN+ tissues. **(E)** Pathway enrichment analyses using Gene Ontology (GO) database. **(F)** Heatmap showing the activated pathways and biological processes among B/Plasma cell subclusters using hallmark gene sets. **(G)** Violin plots showing the different levels of activated pathways and biological processes between LN- and LN+ tissues in each B/Plasma cell subcluster. **(H)** Cellchat analyses showing number of interactions among different cell types separately in LN- and LN+ tissues. **(I)** Comparison of the number and strength of inferred interactions in LN- and LN+ tissues.

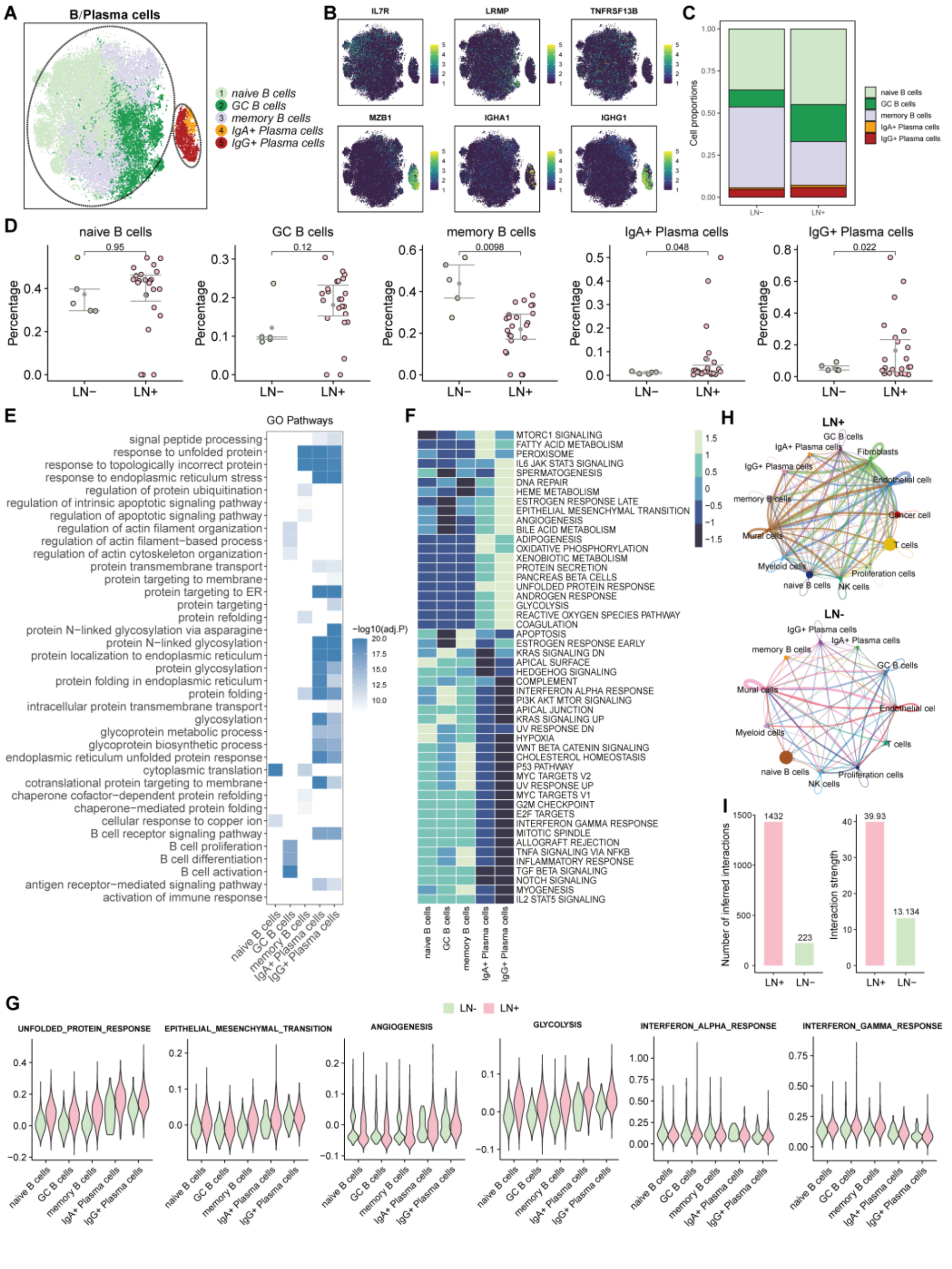

Supplementary Figure S6. Discernment of common expression programs of cancer cells in breast cancer LN metastatic lesions and identified *GLO1* was a biomarker of LN metastasis in breast cancer. (A) Pathway enrichment analyses in each module using hallmark signatures. **(B)** Box plot showing the expression of *CRABP2* and *ERBB2* between normal and tumor tissues in TCGA-BRCA dataset. **(C)** Violin plots showing *CRABP2*, *ERBB2*, and *GLO1* expression levels between LN- and LN+ tissues in each major cell type. **(D)** Violin plots showing *GLO1* expression levels among breast cancer subtypes. **(E)** Violin plots showing *GLO1* expression levels among each cell type in ST datasets. **(F)** K-M survival analysis showing the specific role of *GLO1* in overall survival, disease-specific survival, disease-free survival, and relapse-free survival outcomes in different cohorts (*** means $p < 0.001$).

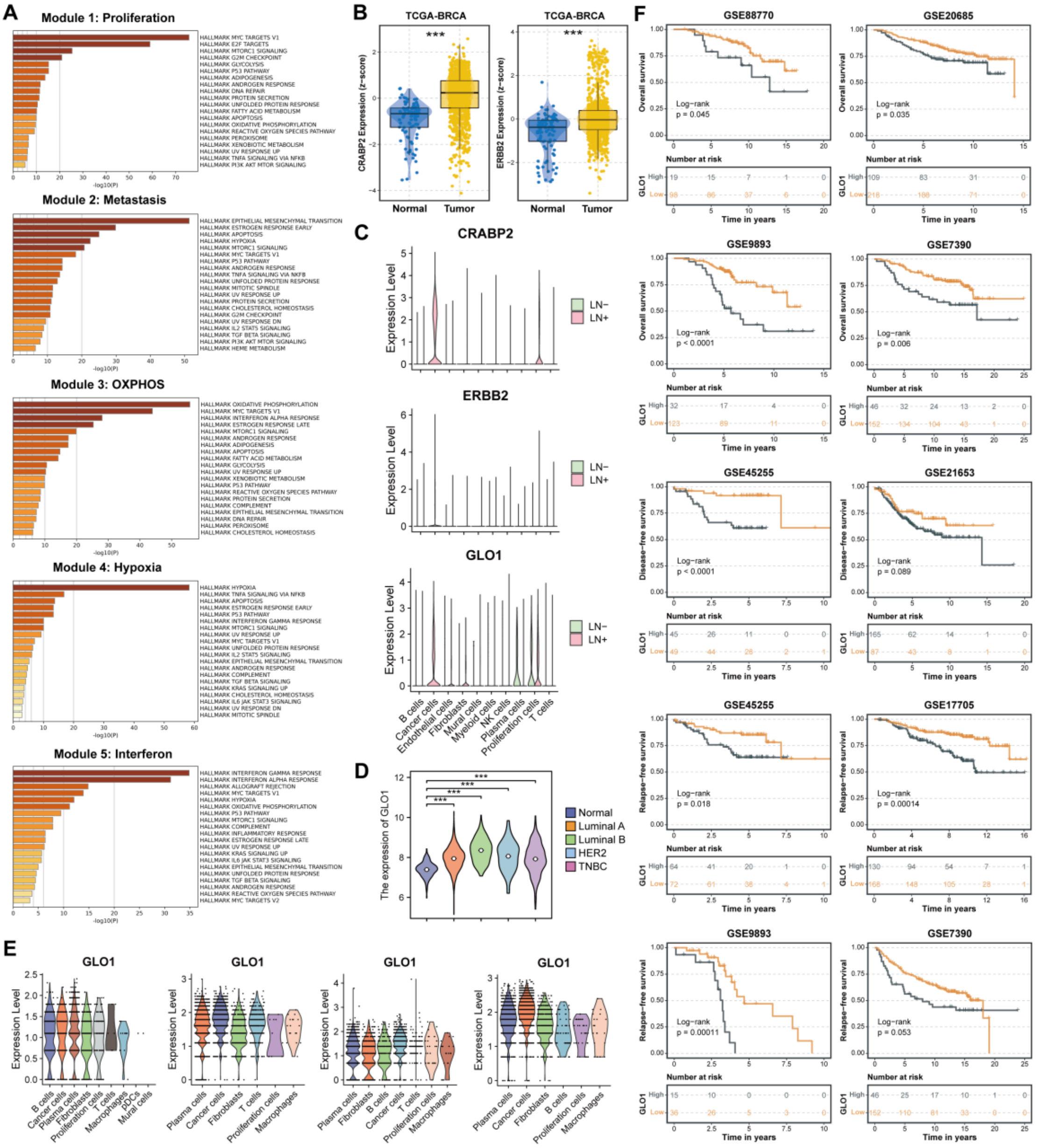

Supplementary Figure S7. *GLO1* promotes lymphangiogenesis and LN metastasis via *VEGFA*-dependent manner and *GLO1* interacts with *GSS*. (A) Correlation ships between *GLO1* and *VEGF* family genes mRNA expression levels using bc-GenExMiner database. **(B)** Box plot showing the expression of *GSS* between normal and tumor tissues in TCGA and GTEx pan-cancer datasets. **(C, D)** qRT-PCR analyses showing the relative *GSS* mRNA levels among different treatments. (ns means no significance, and *** means p < 0.001).

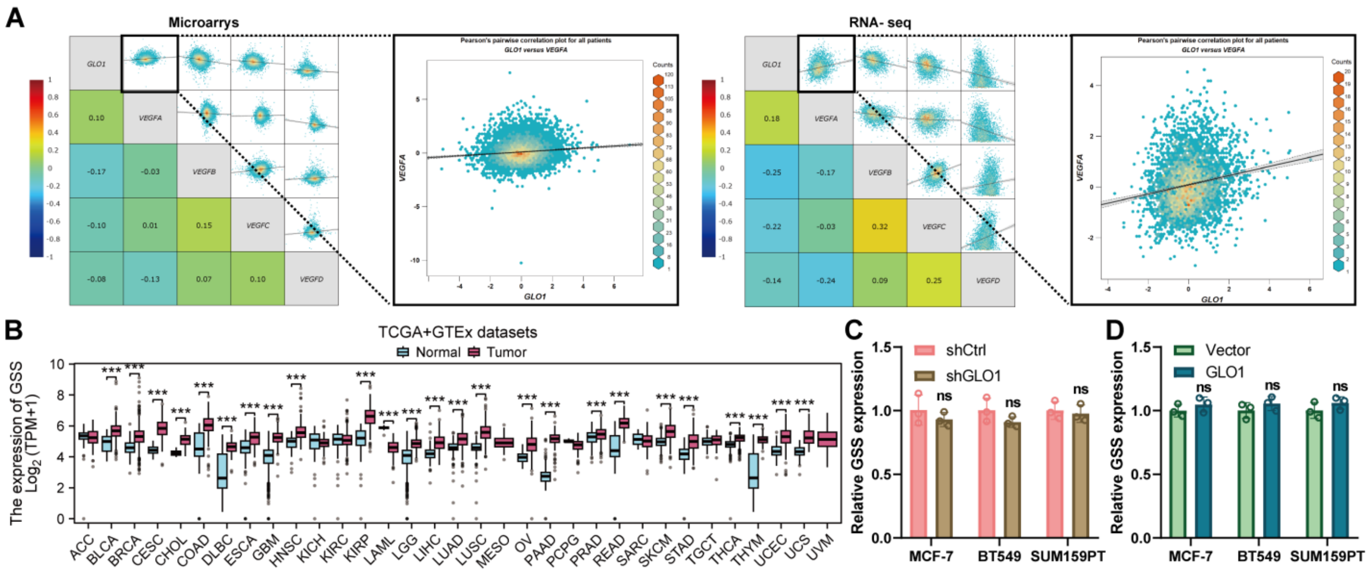

Supplement: Supplementary file 1 — Supporting Information [file ADVS-12-e00722-s002.pdf]
