# Supplementary material for: Paracrine Orchestration of Tumor Microenvironment Remodeling Induced by GLO1 Potentiates Lymph Node Metastasis in Breast Cancer
Source: Adv Sci (Weinh). 2025 Jun 10;12(32):e00722. doi: 10.1002/advs.202500722 (PMC12407353; doi:10.1002/advs.202500722)
Supplement: Supplementary file 2 — Supporting Information [file ADVS-12-e00722-s001.pdf]

ADVANCED SCIENCE
Open Access

Supporting Information

for *Adv. Sci.*, DOI 10.1002/advs.202500722

Paracrine Orchestration of Tumor Microenvironment Remodeling Induced by GLO1 Potentiates Lymph Node Metastasis in Breast Cancer

Jindong Xie, Wenjian Liu, Xinpei Deng, Huan Wang, Xueqi Ou, Xin An, Min-Yi Situ, Anli Yang, Chuan Peng, Rongfang He, Yi Xie, Hailin Tang, Yuman Chen, Jie-Ying Liang, Ruonan Shao, Weikai Xiao* and Shaoquan Zheng**

Supplementary Table S1. Clinical information for samples analyzed by scRNA-seq in this study.

Patient	Gender	Cohort	LN status	Subtype	Tumor Size (mm)
Sample 1	Female	GSE161529	Metastasis	Luminal	17
Sample 2	Female	GSE161529	Metastasis	Luminal	-
Sample 3	Female	GSE161529	Metastasis	Luminal	12
Sample 4	Female	GSE161529	Metastasis	Luminal	25
Sample 5	Female	GSE161529	Metastasis	Luminal	25
Sample 6	Female	GSE161529	Metastasis	Luminal	13
Sample 7	Female	GSE167036	Metastasis	HER2+	12
Sample 8	Female	GSE167036	Metastasis	Luminal	20
Sample 9	Female	GSE167036	Metastasis	Luminal	20
Sample 10	Female	GSE167036	Metastasis	Luminal	13
Sample 11	Female	GSE167036	Metastasis	Luminal	20
Sample 12	Female	GSE167036	Metastasis	Luminal	18

Sample 13	Female	GSE167036	Metastasis	HER2+	22
Sample 14	Female	GSE167036	Metastasis	HER2+	21
Sample 15	Female	GSE180286	Non-metastasis	TNBC	-
Sample 16	Female	GSE180286	Non-metastasis	TNBC	-
Sample 17	Female	GSE180286	Metastasis	Luminal	-
Sample 18	Female	GSE180286	Non-metastasis	Luminal	-
Sample 19	Female	GSE180286	Metastasis	HER2+	-
Sample 20	Female	GSE180286	Non-metastasis	HER2+	-
Sample 21	Female	GSE180286	Metastasis	TNBC	-
Sample 22	Female	GSE180286	Metastasis	TNBC	-
Sample 23	Female	GSE180286	Metastasis	HER2+	-
Sample 24	Female	GSE180286	Non-metastasis	HER2+	-
Sample 25	Female	GSE225600	Metastasis	Luminal	-
Sample 26	Female	GSE225600	Metastasis	HER2+	-

Sample 27	Female	GSE225600	Metastasis	Luminal	-
Sample 28	Female	GSE225600	Metastasis	Luminal	-

Supplementary Table S2. siRNA sequences.

siCtrl	UUCUCCGAACGUGUCACGUTT
siACTA2	GGACCUCUAUGCUAACAAUTT
siVEGFA	GGGCCUCCGAAACCAUGAATT
siGSS	CUGUGCAGAUGGACUUCAATT

Supplementary Table S3. shRNA sequences.

shCtrl	TTCTCCGAACGTGTCACGT
shGLO1	GAAACCTGATGATGGTAAA

Supplementary Table S4. Primers for qRT-PCR detection.

GLO1	Forward	AAGCAGGCTAGGCATGTGAA
	Reverse	CCCAAGAGCCAAGAGCACAA
GSS	Forward	CCCTAGCCGGTTTGTGCTAA
	Reverse	GCCTGTACCATTTCCTCCCC
β-actin	Forward	CATGTACGTTGCTATCCAGGC
	Reverse	CTCCTTAATGTCACGCACGAT

Supplementary Table S5. 18 common proteins possibly interact with GLO1 assessed by mass spectrum in breast cancer cell lines (BT549, MCF-7, and SUM159PT).

Protein ID	Protein description	Gene symbol
sp|Q9NQX3|GEPH_HUMAN	Gephyrin	GPHN
sp|Q13561|DCTN2_HUMAN	Dynactin subunit 2	DCTN2
sp|P57678|GEMI4_HUMAN	Gem-associated protein 4	GEMIN4
sp|Q9UHI6|DDX20_HUMAN	Probable ATP-dependent RNA helicase DDX20	DDX20
sp|Q8N163|CCAR2_HUMAN	Cell cycle and apoptosis regulator protein 2	CCAR2
sp|O15145|ARPC3_HUMAN	Actin-related protein 2/3 complex subunit 3	ARPC3
sp|O75531|BAF_HUMAN	Barrier-to-autointegration factor	BANF1
sp|Q14203|DCTN1_HUMAN	Dynactin subunit 1	DCTN1
sp|Q16637|SMN_HUMAN	Survival motor neuron protein	SMN1
sp|P62899|RL31_HUMAN	Large ribosomal subunit protein eL31	RPL31
sp|P0C0S8|H2A1_HUMAN	Histone H2A type 1	H2AC11
sp|P62318|SMD3_HUMAN	Small nuclear ribonucleoprotein Sm D3	SNRPD3
sp|O43242|PSMD3_HUMAN	26S proteasome non-ATPase regulatory subunit 3	PSMD3
sp|P62314|SMD1_HUMAN	Small nuclear ribonucleoprotein Sm D1	SNRPD1
sp|P40925|MDHC_HUMAN	Malate dehydrogenase, cytoplasmic	MDH1
sp|P25398|RS12_HUMAN	Small ribosomal subunit protein eS12	RPS12
sp|P14678|RSMB_HUMAN	Small nuclear ribonucleoprotein-associated proteins B	SNRPB
sp|P48637|GSHB_HUMAN	Glutathione synthetase	GSS
